# Roles for Pathogen Interference in Influenza Vaccination, with Implications to Vaccine Effectiveness (VE) and Attribution of Influenza Deaths

**DOI:** 10.3390/idr14050076

**Published:** 2022-09-23

**Authors:** Rodney P. Jones, Andrey Ponomarenko

**Affiliations:** 1 Healthcare Analysis and Forecasting, Wantage OX12 0NE, UK; 2 Department of Biophysics, Informatics and Medical Instrumentation, Odessa National Medical University, Valikhovsky Lane 2, 65082 Odessa, Ukraine

**Keywords:** influenza, vaccination, pathogen interference, virus interference, vaccine effectiveness, spatiotemporal variability, influenza-like illness, age, vaccination coverage, pathogen burden, persistent pathogens

## Abstract

Pathogen interference is the ability of one pathogen to alter the course and clinical outcomes of infection by another. With up to 3000 species of human pathogens the potential combinations are vast. These combinations operate within further immune complexity induced by infection with multiple persistent pathogens, and by the role which the human microbiome plays in maintaining health, immune function, and resistance to infection. All the above are further complicated by malnutrition in children and the elderly. Influenza vaccination offers a measure of protection for elderly individuals subsequently infected with influenza. However, all vaccines induce both specific and non-specific effects. The specific effects involve stimulation of humoral and cellular immunity, while the nonspecific effects are far more nuanced including changes in gene expression patterns and production of small RNAs which contribute to pathogen interference. Little is known about the outcomes of vaccinated elderly not subsequently infected with influenza but infected with multiple other non-influenza winter pathogens. In this review we propose that in certain years the specific antigen mix in the seasonal influenza vaccine inadvertently increases the risk of infection from other non-influenza pathogens. The possibility that vaccination could upset the pathogen balance, and that the timing of vaccination relative to the pathogen balance was critical to success, was proposed in 2010 but was seemingly ignored. Persons vaccinated early in the winter are more likely to experience higher pathogen interference. Implications to the estimation of vaccine effectiveness and influenza deaths are discussed.

## 1. Background

This review represents the fourth part in a series on the determinants of excess winter mortality [1,2,3]. In the first part, excess winter mortality (EWM) was shown to be directly measurable using monthly deaths (all-cause mortality). EWM is the percentage difference between four ‘winter’ months and eight ‘non-winter’ months [1]. In the early 1900s ‘winter’ generally occurred later than in recent times [2]. Such data are readily available for around 120 world countries [1], and at state/province level in many other countries. System complexity theory was then invoked to explain the long-term cycles in EWM seen over the past century [2]. It was noted that while the 1918–1919 Spanish flu pandemic did indeed lead to very high EWM, all subsequent flu pandemics showed an EWM which was within the range for ‘ordinary’ seasonal influenza [2]. Antigenic distance between the vaccine and the circulating wild type variants in each location was the most important factor influencing the efficacy of the vaccine [2]. Over-counting of estimated influenza deaths was demonstrated [2]. The next study demonstrated that influenza vaccination was associated with increased total mortality in around 40% of winters and that the long-term average benefit from influenza vaccination over a 40-year period was a mere 0.3% reduction in excess winter mortality at a theoretical 100% vaccination rate in the elderly [3]. In a single pathogen world such an outcome would be illogical, however, we live in a highly complex multi-pathogen world where system complexity theory shows that well intended interventions can lead to unexpected outcomes. The mechanisms behind pathogen interference were suggested as a potential source of the unanticipated increased mortality observed in some winters [3].

This review explores the potential for pathogen interference, the mechanisms by which it is expressed, and how the variable antigenic mix in seasonal influenza vaccines may inadvertently lead to adverse outcomes in particular years.

We do not in any way question the fact that influenza vaccination offers a measure of protection against influenza induced death [4,5], howbeit, such protection is somewhat mediocre in the elderly—average vaccine effectiveness is only 40% [6]. We accept this as an established fact; however, winter deaths are far wider than just influenza(s) and there is a mounting body of evidence that influenza vaccination, per se, can trigger an unexpected shift in pathogen interference. This review will examine both the direct and indirect evidence for this position.

This review is not intended as a systematic review of pathogen interference per se, but to explore if influenza vaccination could trigger unexpected outcomes within the context of pathogen interference. The aim is to explore fundamental principles which explain why studies conducted in different places and at different times appear to give conflicting results. We commence with an overview of the wider context of pathogen interference and then move to the specifics relating to respiratory infections and the potential unintended effects of influenza vaccination within a bigger picture of high system complexity.

## 2. Introduction

Pathogen interference, namely, one pathogen altering the infection and/or expressed pathology by another is well known in plants [7], insects [8,9], birds [10], fish [11] and wider animal kingdom [12]. As is to be expected pathogen interference also operates in humans [13,14,15,16] and includes altered clinical outcomes in coinfections and superinfection [16]. Many of these interactions are mediated by small or microRNAs (miRNAs) which can also interfere with host immune defenses [17,18,19,20,21]. miRNAs are also involved in epigenetic mechanisms including gene silencing [22]. Viral interference can also be mediated by factors such as interferons (IFNs), defective interfering (DI) particles, production of trans-acting proteases, cellular factors, and nonspecific double-stranded RNA (dsRNA) [23,24]. The review of Kumar et al. [16] gives a long list of coinfections leading to accommodation, interference, and enhancement. Exchange of genetic material during mixed infections of animals and humans by influenza(s) acts to create influenza virus diversity including pandemic strains [25].

The response of the host immune system also influences the outcome of viral coinfections. Upon antigen exposure, naive T cells convert into activated effector T cells and eventually into memory T cells. The memory responses generated against one infection may influence the quantity and quality of the immune response to subsequent primary or secondary infection(s). This influence of immunity to primary infection on a subsequent unrelated infection is known as heterologous immunity. Heterologous immunity can occur between very closely related infectious agents such as multiple variants of a particular virus type, among different viruses, or between viruses, bacteria, protozoa, or different parasites. A variety of immune cells participate in heterologous immunity, and these may induce either a protective or immunopathological response [24].

Winter mortality, influenza infection and vaccination form a multidimensional complex problem [2,3]. Here, we detail the complex components within a structured framework regarding the role of pathogen interference and its potential impact upon the net effects of influenza vaccination, upon the measurement of vaccine effectiveness (VE) and upon the estimation of influenza deaths.

## 3. A Wider Context to Pathogen Interference

### 3.1. How Many Human Pathogens

Accepting the fact that pathogen interference is a common phenomenon it is sensible to explore exactly how many human pathogens have been detected. Table 1 presents the results of two such estimates made in 2005 and 2012. In just 7 years the total number of species had increased by 50%, with a notable 86% increase in bacterial species.

A study published in 2012 [28], established that new species of human viruses were being discovered at a rate of around 4 per year, and it was estimated that a further 265 remained to be discovered (range 89 to >2000) [28]. This estimate would give somewhere >310 species of human viruses by 2022.

One study conducted in Salt Lake City, Utah, USA which was specific to the detection of bacteria in 26,000 clinical samples collected between 2006 and 2010 revealed 111 novel genera and 673 potentially novel species [29]. Hence, in 2012 this would take the number of clinically significant bacterial species to >1676, and probably >2000 by 2022.

This potential for undiscovered species was further illustrated in a 2021 study which analyzed 4728 samples from the surfaces of urban transport systems (railings, benches, ticket kiosks, etc.) in 60 world cities [30]. The samples were analyzed for the DNA of the microbes on these surfaces. RNA viruses such as influenza or coronavirus (SARS, MERS, COVID-19) were not included in the study. Some 4236 known species of DNA-based microbes were identified plus an additional 10,929 new DNA viruses and 1302 new species of bacteria. For every 10 additional samples another new species was identified [30]. While many of these microorganisms will be from soil, air, feces (human and otherwise) and contact with animals, some will be from the commensal human microbiome (especially the skin), and some will be potential pathogens.

Regarding the RNA viruses, a study published in 2022 involving 5.7 million biological samples identified over 100,000 novel RNA viruses in addition to those already known [31]. Once again, only some of these will be direct human pathogens, however, the potential magnitude of the situation is evident.

Table 1 shows an estimate for the number of known human pathogens by 2022 assuming that the rate of discovery is slowing over time. Hence, the total potential for pathogen interference is vastly more complex than the current limited number of human studies using common pathogens. Below species level there will be tens of thousands of strains and variants, each of differing clinical importance.

### 3.2. Pathogen Burden and Persistent Infections

Pathogen burden is a count of antibodies to pathogens to which a person has been exposed. A large proportion of the pathogen burden is due to persistent or intracellular infections—which is a subset of the total number of human pathogens in Table 1. They maintain a persistent infection by manipulating diverse aspects of host immune function [32]. The ensuing background level of multiple layers of immune manipulation have been linked to chronic mental and physical diseases including autoimmunity [33,34,35,36,37], increased speed of ageing [38,39,40,41], and chronic inflammation [42]. Pathogen burden is highest among the most disadvantaged social groups [43]—who also experience lower lifespan.

Pathogen interference therefore operates within the wider context of immune function manipulation by the pathogen burden.

Table 1 covers all countries and all human pathogens so far detected; however, how many pathogens do humans commonly encounter? One study regarding DNA viruses in healthy humans discovered an average of 5.5 species per individual with a maximum of 15 in one individual [44]. Given the fact that this is just DNA viruses the total pathogen burden and its range will be considerably higher.

### 3.3. Roles for the Immune Modifying Persistent Virus Cytomegalovirus

Cytomegalovirus (CMV) is a common herpes virus with one of the largest viral genomes enabling it to exert a formidable array of immune manipulative strategies [45,46]. The genome codes for 4 noncoding RNAs and 14 miRNAs [47]. CMV can infect a wide range of cell types [48]. It is both oncogenic and oncomodulatory [49], implicated in autoimmune diseases [36,37], and consistently appears in studies relating to mental [35] and physical health [39,50,51,52,53,54,55], and the reduction in lifespan [38,41,53,56]. CMV appears to work mainly by manipulation and stealth rather than by overt infection [45,46]. It is often one of the joint pathogens involved in the expression of pathogen burden.

CMV exploits interferon-induced transmembrane proteins (IFITMs) which are also regulated by miRNAs [57]. Direct interference of infection by RNA viruses is likely. This is especially relevant to influenza since the lung is a major reservoir for CMV infection [50]. CMV was proposed to have deleterious effects upon around 20% of the population [50]. Reactivation of CMV is more common in old age and during periods of immune erosion [47]. CMV has also been linked to poor response to influenza vaccination, however, this appears to depend on the vaccine antigenic mix [53]. This is largely unexplored territory but suggests that the intricacies of pathogen interference via wider immune function may be more complex than appreciated, especially in persons infected with CMV.

### 3.4. Heterologous Immunity

Heterologous immunity is the ability of one pathogen to alter the immune response to and outcome of infection by a subsequent pathogen [24,58,59,60,61]. It represents a sub-set of pathogen interference. Heterologous immunity is also proposed to be involved in drug hypersensitivity [62]. Since vaccination acts as a proxy infection, heterologous immunity is of profound importance in vaccine development and real-world efficacy [57,63,64].

Heterologous immunity provides a basis for some of the unanticipated outcomes of influenza vaccination previously observed [3]. However, this is a poorly studied area.

### 3.5. Antigenic Original Sin and Immune Priming

Antigenic original sin or immune priming is the process whereby the immune system mounts a response against the first example of an infection by a pathogen [65,66]. If there is sufficient antigenic similarity this acts to curtail the infection, however, when the necessary similarity is absent this can lead to a futile immune response causing enhanced infection and poor clinical outcomes [67,68].

This is especially the case for influenza(s) [65] and in influenza vaccine response [69]. This represents another aspect of wider pathogen interference and resulting clinical outcomes. The overall picture is one of an increasingly complex host–pathogen immune framework.

### 3.6. Immune Function and Malnutrition in Children and the Elderly

All the above operates within the immune consequences of malnutrition especially in children and the elderly [70], leading to the profound diversity seen in human immune function and parameters [70,71,72].

### 3.7. Role of the Human Microbiome

The human microbiome within the skin, digestive tract, respiratory tract, and reproductive tract are complex microbial communities with an important role in the maintenance of overall physical and mental health, and are extensively involved in pathogen-related diseases [32,33,34,35,36,37,38,39,40,41,42,43,44,45,46,47,48,49,50,51,52,53,54,55,56,57,58,59,60,61,62,63,64,65,66,67,68,69,70,71,72,73,74,75,76,77,78,79]. These microbiomes seemingly communicate with each other and the immune system [79]. While the protective role of probiotic bacteria in the gut is widely appreciated [80,81], the role of the respiratory tract microbiome is equally important.

Studies have demonstrated that the pharyngeal microbiome comprises many bacterial species that interact with the local epithelial and immune cells, forming a unique micro-ecological system. Most are obligate symbionts constantly adapting to their unique surroundings. Indigenous commensal species both maintain dominance and evoke host immune responses to eliminate invading species. Temporary damage due to the impaired local epithelia is also considered an important predisposing risk factor for infections [74].

Using a household transmission study, Lee et al. [82] examined whether the nose/throat microbiota was associated with influenza susceptibility. Five bacterial community types were identified. One nasal/oropharyngeal community state type (CST) was associated with decreased susceptibility to influenza. This CST was rare and transitory among young children but prevalent and stable among adults. Associations between the nose/throat microbiota and influenza also existed at the taxa level, specifically with the relative abundance of Alloprevotella, Prevotella, and Bacteroides oligotypes. High rates of change between bacterial CST among both secondary cases and household contacts who were not infected were also identified. Most importantly age was strongly associated with susceptibility to influenza and the nose/throat bacterial community structure [82]. 

Another household transmission study recruited 115 index cases with influenza A(H3N2) or B infection and 436 household contacts [83]. A 10-fold increase in the abundance in Streptococcus spp. or Prevotella salivae was associated with 48% and 25% lower respective susceptibility to influenza A(H3N2) infection. For influenza B infection, a 10-fold increase in the abundance in *Streptococcus vestibularis* or *Prevotella* spp. was associated with 63% lower and 83% respective higher susceptibility [83].

Regarding shedding of influenza another study showed that among secondary cases of influenza, higher bacterial community diversity before infection was associated with 60% longer shedding duration and earlier time to infection. Neisseria and multiple other oligotypes were significantly associated with symptom and shedding durations and time to infection [84].

The respiratory microbiota and communication between the gut and respiratory microbiota are directly affected by malnutrition [85]. The role of the respiratory microbiota in pathogen interference remains unexplored.

### 3.8. Influenza (and Other Pathogens) Show Extreme Spatiotemporal Variation

It is widely recognized that infectious outbreaks show extreme spatiotemporal variation (space–time variation) both between countries and within a country [86,87,88,89,90,91]. As an example, the winter of 2014/15 saw high international excess winter mortality, partly due to the emergence of new influenza A/H1N1 clade(s) [3].

The UK was badly affected, however, while 69% of 512 local government areas reached a maximum excess winter mortality (EWM) in March 2015 the other 31% ranged from December 2014 to April 2015 and the interquartile range for maximum EWM was from 25% to 34% (calculation based on data sources in [1,2]). Lowest EWM in that winter was only 7.2% in Great Yarmouth (Norfolk), 8% in Tamworth (Staffordshire), and 8.8% in Omagh (Northern Ireland). To some extent meteorological variables, mainly temperature, humidity, rainfall, weather systems and their instability, seem to be implicated although the relative importance of these variables changes between the tropical and temperate regions [92,93,94,95,96,97,98]. However, the sheer extent of the spatiotemporal variability within the UK seems to imply that other important non–meteorological factors are also involved.

**Figure 1:**
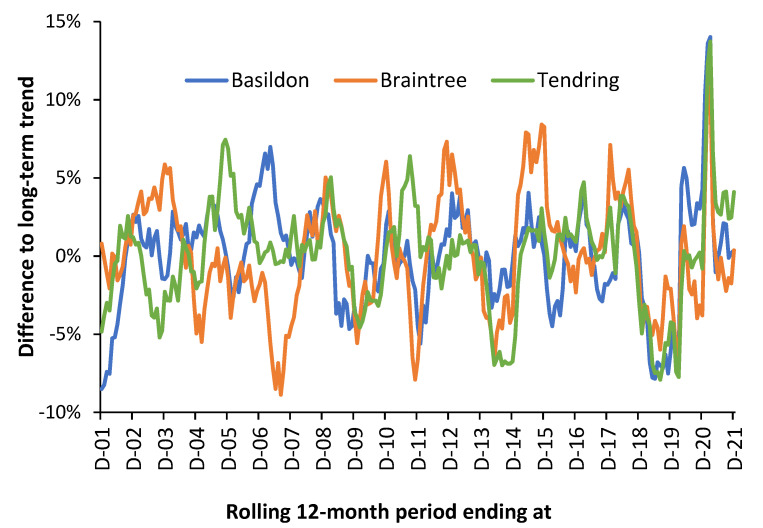
Percentage difference between the running/rolling 12-month total of monthly deaths and the long-term trend for three local authorities in the county of Essex, East of England, 2001 to 2021. The underlying/long-term trend was determined by a second order polynomial curve fit. The total number of deaths increases over time. Data are from the Office for National Statistics in [21,91].

This is illustrated in Figure 1 using three local authorities in the county of Essex in the East of England using a rolling/moving 12-month total (sum) of all-cause deaths. The rolling 12-month total starts at December 2001, move forward by one month and recalculate. The whole of Essex only encompasses 830 square miles. Given the proximity of these local authorities it is highly unlikely that meteorological variables explain the differences. Recall that each point on this chart is a 12-month total (sum) which should remove underlying seasonality.

In Figure 1 the maximum Poisson standard deviation associated with the trend is ±2.7% for Braintree in 2001 falling to ±2.4% in 2021. The other local authorities are lower than this due to larger size. Hence, the trend is dominated by systematic variation rather than random variation. Especially note the extreme divergence during 2007 and 2011. The large spike in the winter of 2020/21 is the second wave of the COVID-19 pandemic. The first wave of COVID-19 was largely absent in Essex.

Note that the shape of the three running 12-month totals tends to preclude the universal importance of a single pathogen, i.e., influenza, and suggests more nuanced multi-pathogen causes. See [99,100,101] for further detail regarding interpreting the shape of a rolling 12-month total chart. It could be observed that while influenza outbreaks appear clear at national level, this is an artifact of divergent small-area behavior.

As an additional comment, age-standardized mortality (ASMR) is a widely calculated measure of population health. However, it is generally calculated, once a year, using calendar year data, i.e., in December in Figure 1, which has been called the ‘calendar year fallacy’ [102]. Figure 1 elegantly shows that the small-area ASMR is therefore largely influenced by the systematic variation. It has been proposed that local mini-outbreaks from the 3000 known and detected species of human pathogens act to precipitate death (often from unrelated conditions) [99,100,101]. Hence, the inexplicable trends seen in Figure 1.

### 3.9. Implications to Pathogen Interference

Section 3 was designed to give a ‘big picture’ view of the multiplicity of immune-microbiome-pathogen interactions lying behind the real-world expression of pathogen interference. This is a prelude to explaining why current pathogen interference studies seemingly do not agree regarding the exact order of pathogen interference. Hence, measured pathogen interference will almost certainly vary by location (region, latitude, microclimate), the time at which the study was conducted, including the time range; and even the individuals in the study (age, gender, inpatient/outpatient). As it were, the bigger context alters the balance between pathogens, which then alters the observed pattern of pathogen interference. These issues will be explored in the next section.

## 4. The Role of Time as a Confounding Variable

**Figure 2:**
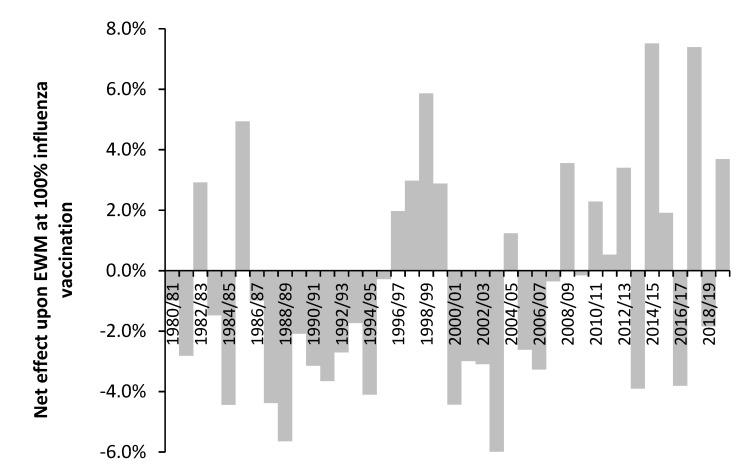
Effect of influenza vaccination upon excess winter mortality (EWM) and age 65+ vaccinated over the period 1980/81 to 2019/20. Footnote: The slope is expressed as the percentage point change in EWM (USA equivalent) at 100% vaccination of the entire population aged 65+. Amount of available data increases over time from 30 in 1980/81 to 74 in 2013/14. From 2005/06 onward there are 69+ data points. The accuracy of the estimated slope increases with time due to higher number of data and a higher range in the proportion vaccinated, i.e., a maximum of only 12% vaccinated in 1988/89, a maximum of 22% vaccinated in 1996/97, rising to a maximum of 51% in 2013/14. The net effect shown in this figure is the average of up to four different methods. The net effect from 1980/81 to 1986/87 only uses one method. Adapted from [3].

Section 3.9 suggested that time may be a confounding variable in the study of pathogen interference and its interaction with influenza vaccination. This proposal is investigated in Figure 2 using the results from the previous study [3] which shows the net effect of influenza vaccination upon excess winter mortality (EWM). If we assume that the net effect of influenza vaccination is moderated by pathogen interference, we can make some tentative inferences about the outcome of pathogen interference studies conducted over multiple years.

Hence, studies conducted over the interval 1986/87 to 1994/95—a period of net benefit from influenza vaccination, could potentially reach a very different conclusion to a study conducted between 1996/97 to 1999/00—a period of net disbenefit from influenza vaccination. A study between 1996/97 and 2003/04 could potentially reach the conclusion that pathogen interference is absent—the average of four beneficial years and four years with net disbenefit. The results from more recent studies are an average of more volatile behavior [3]. Note that the net effect shown in Figure 2 is an ‘international’ average, and that individual countries deviate from this average in particular years.

The results of some studies have questioned any role for pathogen interference upon influenza vaccine effectiveness; however, such studies were seemingly conducted over periods when the average effect of influenza vaccination was close to zero.

## 5. Respiratory Pathogen Interference

The following sections will discuss respiratory pathogen interference and potential interactions with influenza vaccination. Due to the highly complex nature of ‘winter’ as a system several key concepts such as the role of weather, age (and nearness to death), and immune function will appear multiple times is different contexts.

### 5.1. An Example of Pathogen Interference and Methodological Issues

The role of pathogen interactions/interference is becoming an area of greater interest [12,13,14,15,16,23,103]. Table 2 presents the results of one study in the context of how common respiratory pathogens may enhance or diminish infection by other respiratory pathogens. Note that the context to this study is shown in the table caption (when, where, who, which pathogens).

Potential interactions with influenza(s) A and B have been highlighted and the relative prevalence of each pathogen is shown—which is specific to the study context. It is assumed that the most frequent pathogens (at the bottom of the table) have the greatest potential to alter mortality in that winter via pathogen interference. The ‘enhanced by’ column should result in a higher frequency of dual infections and superinfection—as in pneumonia after influenza (discussed later in Section 8.2).

Given the fact that persistent pathogens have not been investigated in this study a secondary layer of potential ambiguity has been added. Since persistent pathogens and the pathogen burden have been ignored in all studies so far conducted on this topic it is unsurprising that differences in order/magnitude exist between studies. Influenza vaccination history is likewise omitted from all studies.

Given this wider context the potential methodological issues surrounding pathogen interference will be briefly summarized. First, is the sampling method, i.e., nasopharyngeal wash, bronchoalveolar lavage (BAL), saliva, sputum, exhaled breath, repeat testing, etc. [103,104,105,106,107,108,109,110]. Different sampling methods are required to optimize the yield of different pathogens [110]. Second is the assay procedure, i.e., cultivation, PCR, or PCR with mass spectroscopy, next generation sequencing, etc. [103,104,105,106,107,108,109,110]. Both will cause differences in apparent prevalence or pathogen load between studies.

Next comes the method of numerical analysis ranging from simple pathogen-pairs (prevalence yes/no) using various statistical tests [103], weekly adjustment for the background prevalence of each pathogen pair compared to actual [111], and a sophisticated multivariate Bayesian framework which included modeling temporal autocorrelation through a hierarchical autoregressive model using the abundance (present yes/no) of the various pathogens [112], and more recently to the examination of pathogen load rather than just yes/no presence [113].

The latest research using pathogen load is that all viruses are mutually adversarial, some more so than others depending on the combination, but that viruses are mutually enhancing of *Streptococcus pneumoniae* infection [113].

Hence, is any method better than others? The optimization of sampling methods specific to each pathogen is a clear priority, as are methods to detect a far wider range of pathogens beyond just the common ones, i.e., DNA/RNA amplification and wider gene libraries. Lastly, many studies just focus on respiratory viruses alone thus ignoring the interplay between viruses and bacteria. The key observation is that the detection of common respiratory pathogens in symptomatic individuals remains very low, typically below 30% [104,112,113,114], which reflects the observations in Section 3 and Table 1 regarding the full range of human pathogens and the role of persistent pathogens—which up to the present has been overlooked in pathogen interference.

Of specific relevance to the potential role of influenza vaccination is the observation that influenza A and B are the most active in inhibiting the load of other viruses [113]. Hence, influenza vaccination is potentially a powerful agent to promote infection by non-influenza viruses, but should be beneficial against *S. pneumoniae* infection, except when it opens the way for non-influenza virus infection.

Clearly, the mix and timing of pathogens is unique to each winter (locality/region/country) [15,113] as is the timing and antigen mix of influenza vaccination in each year [88]. It is the antigen mix of each seasonal influenza vaccination [88,89] which most likely imposes a degree of international commonality observed in the previous study [3].

The largely ignored paper published in 2010 by a group of Hungarian researchers is of great relevance to the issues surrounding the potential unintended effects of influenza vaccination and pathogen interference [15]. They noted that the timing of vaccination with respect to levels of key pathogens could enhance or diminish vaccine effectiveness (VE), and that vaccination (in general) could enhance the circulation of certain pathogens [15]. Section 8 will explore potential immune mechanisms for the unintended effects of influenza vaccination in a world of competing pathogens.

### 5.2. How Common Is Influenza Infection

In the USA it has been estimated that between 3% to 11% of the population show evidence for symptomatic influenza infection in different years [91]. A study using 18 years of data concluded that influenza infection rates decline with age down to an average of 8% per annum at age 70 [114].

Around 60 million people were estimated to die in 2020 [115]. Some 20 million will die each winter or winter equivalent in the tropics. Hence, there is ample scope for influenza and wider pathogen interference to affect winter mortality. Although a recent study suggests that true influenza-attributable deaths appear to have been substantially over-estimated [2], which may partly be due to the difficulty of separating ‘with’ influenza from ‘due to’ influenza as the cause of death. This same problem has plagued the reporting of COVID-19 deaths [116]. Incorrect attribution of non-influenza deaths to influenza is also possible.

The point of relevance is that large numbers of persons typically receive influenza vaccination each year while only 3% to 11% of these have a symptomatic influenza infection. What happens in the approximate 90% after receiving influenza vaccination; who do not experience an influenza infection, but contract a relatively more common non-influenza infection?

### 5.3. Pathogen Interference and Influenza-Like Illness (ILI)

Public health agencies around the world commonly use the levels of ILI or acute respiratory infection (ARI) as a measure of the incidence of influenza each winter. Some 100 pathogens can cause symptoms of ILI [117]. Along with influenza viruses A and B, parainfluenza virus, respiratory syncytial virus (RSV), adenovirus and *Mycoplasma pneumoniae* are regarded as important respiratory pathogens with the potential to cause ILI [118]. *Streptococcus pneumoniae* (pneumococcus) and Haemophilus influenzae type b were identified as the main bacterial causes of pneumonia (sometimes as a complication of influenza infection) while RSV and hMPV were considered the most prevalent viral causes [119]. Coinfection is common [103,120] and was observed in 24% of ARI cases in Table 2.

Hence, one study used influenza A/H1, A/H3, B, RSV, and human parainfluenza virus types 1, 2, 3 to derive better forecasts of ILI [121]. During a large ILI outbreak in New York in 2004/05 a new genetic clade of rhinovirus was identified [122]. ILI rates correlate poorly with winter deaths. In England, the highest ILI rate of 150 cases per 100,000 population occurred in week 30 of 2009 (during the Swine flu pandemic) when there were only baseline levels of deaths, while the highest excess deaths occurred in weeks 1 and 2 of 2015 when there were only just over 20 ILI cases per 100,000 which is only slightly above the seasonal threshold for an ‘epidemic’ [100].

In the context of this study, note the relatively low prevalence of influenza (only 11.2%) in the nasopharyngeal samples of Acute Respiratory Infection (ARI) patients in Table 2 [103]—in what was an average EWM winter. Only one of the 21 tested pathogens was detected in 42% of samples. We highlight the fact that none of the 21 common pathogens were detected in 32% of the ARI patients [103]. In an eight–year study (2004/05 to 2012/13) for persons with ARI only 14% tested positive for influenza A and 9% for influenza B [123]. It is common for less than 20% of ILI samples to test positive for influenza [124,125,126]—as also observed for ARI in Table 2.

The proportion of influenza may be higher in adults aged 60+ [127] and in epidemic–level influenza. In England, samples submitted from primary care settings for persons presenting with ILI for influenza confirmation are generally below a maximum of 30–35% positive [128,129,130,131,132,133]. A review suggested that around 25% was common [134] and concluded that ILI was not an appropriate measure for influenza activity or vaccine effectiveness. It was noted that no pathogen (at least among those tested) was identified in up to 50% of influenza negative samples [134]. This merely confirms the fact that multiple organisms cause ILI symptoms of which about 30% to 50% are uncommon pathogens. Hence, ILI is regarded as a poor measure of the true VE for influenza or as an indicator of influenza seasonal severity [127,134].

A large study of hospitalized patients with ILI or pneumonia showed that among adults 55% had non-influenza respiratory virus (NIRV) infections (hRV 14.9%, RSV 12.9%, hCoV 8.2%). Overall, 15% of NIRV infections were acquired in hospital. Admission to ICU, hospital length-of-stay, and 30-day mortality were similar for patients with NIRV infection and those with influenza. Age > 60 years, immunocompromised state and hospital-acquired viral infection were associated with worse outcomes. The estimated median cost per acute care admission for any respiratory pathogen was $6000 (IQR $2000–$16,000) [135]

All the above is unsurprising given the fundamental fact that ILI is the by–product of the production of interferons (and other cytokines), as pathogens seek to limit coinfection by other pathogens and promote wider immune responses [136]. We propose that ILI is more a measure of the net pathogen interference than of influenza prevalence per se.

Regarding mortality from non-influenza respiratory viruses (NIRV), a study in Canada concluded that “the burden of NIRV infection is substantial in adults admitted to hospital and associated outcomes may be as severe as for influenza” [135]. Such observations confirm our findings that influenza mortality is likely to have been substantially overestimated [2].

### 5.4. Pathogen Interference, Influenza Infection and Vaccination

Implicit in the estimation of influenza vaccine effectiveness [VE] is the assumption that vaccination has no effect on pathogen interference [137]. However, Opatowski et al. [14] reviewed the evidence for influenza/non-influenza pathogen interactions with a view to modelling the effects upon influenza pathogenesis and epidemic profiles. This study implies that pathogen interference could alter VE. The immune responses regulating VE and pathogen interference may well be very different and will be discussed later.

In a study involving children and adolescents’, prior inactivated trivalent influenza vaccination (TIV) in 2008/09 was demonstrated to reduce the risk of subsequent human coronavirus infection [138]. Interestingly, prior influenza vaccination has also been shown to reduce the risk of COVID–19 illness and severity [139,140]. If influenza vaccination can diminish infection by another species, then the reverse must also apply.

Of relevance is a randomized trial involving 115 children using trivalent inactivated influenza vaccine (TIV) or placebo during the 2008/09 influenza season where the vaccine did protect against influenza–confirmed illness [141]. However, TIV recipients had an increased risk of non–influenza ARI (RR: 4.4–times higher (CI 1.3–14.8) and of virologically confirmed non–influenza infections (RR: 3.5–times higher, CI 1.2–10.1). The authors suggested that in protection against influenza, TIV recipients may lack temporary non–specific immunity that protected against other respiratory viruses [141].

A study (70% children) over three influenza seasons (2013–2016) showed that children but not adults were at higher risk of non–influenza pathogens (including 3 bacteria) and that this occurred in both the 14 days post vaccination and beyond. There were very few adults aged 50+ in this study [142].

A study among US military personnel and their families gave mixed results regarding influenza vaccination in the 2017/18 season and consequent non–influenza viral infection. This study had some deficiencies in that only a small proportion of the military personnel are not vaccinated, and most unvaccinated individuals were their children. All confidence intervals overlapped. There was some suggestion that virus interference may be present in the military personnel although the confidence interval was very wide. As expected, the odds for influenza infection were lower in the vaccinated group [143].

A study over two consecutive influenza seasons (2011/12 and 2012/13) for adults aged 60+ demonstrated that influenza vaccination reduced the incidence of influenza infection (VE of 73% and 51%, respectively) in patients exhibiting ILI [127]. However, the overall rate of ILI was not reduced by influenza vaccination because influenza was substituted by other pathogens. As expected, the proportions of the other pathogens were season specific [127]. During the two years of this study the net effect of influenza vaccination was for a 2% increase in EWM at 100% aged 65+ vaccination [3], i.e., pathogen interference could be assumed to be operative in these years.

Regarding the apparent lack of a response to influenza vaccination observed above in adults, a study of military recruits is relevant [144]. Military recruits experience a high incidence of febrile respiratory illness (FRI), leading to significant morbidity and lost training time. Adenoviruses, Streptococcus pyogenes (group A), and influenza virus are implicated in over half of the FRI cases reported at recruit training center clinics. Analysis of FRI cases showed that rhinoviruses, adenoviruses, *S. pneumoniae*, *H. influenzae*, and *N. meningitidis* were widely distributed in recruits. Of these five agents, only adenovirus showed significant correlation with illness. Among the samples tested, only pathogens associated with FRI, such as adenovirus 4 and enterovirus 68, revealed strong temporal and spatial clustering of specific strains, indicating that they are transmitted primarily within sites (as implied in Figure 1). A strong negative association between adenoviral FRI and the presence of rhinoviruses in recruits, suggesting some form of viral interference [144]. Adults seemingly experience higher rates of infection by a different range of pathogens to children and the elderly. Indeed, the study investigating pathogen load noted unique age profiles for each pathogen [113].

Hence, children and the elderly appear susceptible to post–vaccination pathogen interference. However, the relationship with age is highly likely to be U– or J–shaped due to the observed reduction in sickness absence among adults of working age [145,146,147], i.e., net ILI (as sickness absence) is reduced in this group.

As mentioned earlier, researchers from diverse locations have reported different outcomes for various pathogen-pair interactions [103,111,112,113] and it should be noted that pathogens such as influenza and RSV have their own unique weather–related forcing parameters [148,149,150,151]. Weather–related patterns in pathogen prevalence will almost certainly explain many of the differences from studies conducted in different locations. A major limitation of most studies is that bacterial infections are either not tested for or are excluded from the study. The study upon which Table 2 was based did include some common bacteria [103].

In conclusion, pathogen interference is highly likely to be adding to the observed high spatiotemporal variation of influenza outbreaks and EWM as observed in Figure 1.

### 5.5. Pathogen Interference and Influenza Outbreaks

With respect to the interaction between influenza and other pathogens an early outbreak of rhinovirus seemingly averted the 2009 Swine flu pandemic in several European countries [152,153,154]—a proposition that agrees with Table 2. This has been confirmed clinically and experimentally with low levels of co–occurrence, and the observation that rhinovirus infected human airway epithelial cells had a 50,000–fold decrease in IAV H1N1pdm09 viral RNA on day 5 post–rhinovirus inoculation [155]. BX795, a drug that blocks innate immune signaling required for the interferon response, restored the ability of influenza to infect the airway cells [155]. Viral interference in airway epithelial cells has its basis in innate immunity and the relative sensitivity of different viruses to various interleukins (IFNs). Inflection with influenza or RSV therefore interferes with rhinovirus replication which is significantly inhibited by IFN–λ and the most sensitive to IFN–α. However, rhinovirus infection does not interfere with influenza or RSV infection [155].

Other studies have demonstrated influenza–virus combinations which occur at low or high frequency [111]. A comprehensive study of 44,230 patients with a respiratory virus infection studied 11 respiratory virus groups over a nine–year period in Scotland. RSV had the most positive (enhances) associations, while rhinovirus and PIV1 had the most negative (diminished by) associations. Influenza A had three positive and two negative associations while influenza B had two positive and two negative associations [112].

It is not widely appreciated that Respiratory Syncytial Virus (RSV) leads to the respiratory–only death of as many aged 65+ as influenza [156,157]. The interaction between influenza(s) and RSV can lead to an alternating pattern of incidence between the two pathogens—although such patterns change with latitude, i.e., weather patterns [147,148,149,150]. In the elderly an RSV infection is often misdiagnosed as influenza [158]. Further detail for RSV is given in Section 8.1. Hence, the high spatiotemporal variation in influenza incidence and EWM is also enhanced by pathogen interference. Given the above the next section will examine the evidence for pathogen interference between COVID-19 and influenza.

### 5.6. Did Lockdown or COVID-19 Halt Influenza in Early 2020

It has been commonly reported that national lockdowns around the world coincided with a dramatic reduction in influenza activity. However, detailed weekly data from the UK disputes this view. COVID-19 began international circulation at some point in late 2019. China reported its first COVID-19 death on 11 January 2020 [159]

In late 2019 there was a modest influenza outbreak in the UK with peak influenza activity and critical care (CCU) admissions at week 51 of 2019 [133]. By the next week (week 52) activity had already suddenly dropped to half this level and CCU admissions had dropped by half by week 2 of 2020. Excess deaths peaked between weeks 49 of 2019 to 2 of 2020, when they temporarily returned to baseline. By week 12 influenza levels were very low and there was no influenza activity and CCU admissions during week 13 onward. Excess deaths began to rise again in week 12 as persons with existing COVID-19 infections were beginning to die in increasing numbers [133].

The intention to implement a national lockdown in the UK was announced on Monday 16 March 2020 (mid-week 12) and was formally announced by the Prime Minister on Monday 23 March but legally came into force on the 26 March (Thursday of week 13) [160].

From this timeline it is evident that influenza activity plummeted around the time COVID-19 infections were taking hold and that influenza had already dropped to zero just before lockdown was implemented.

**Figure 3:**
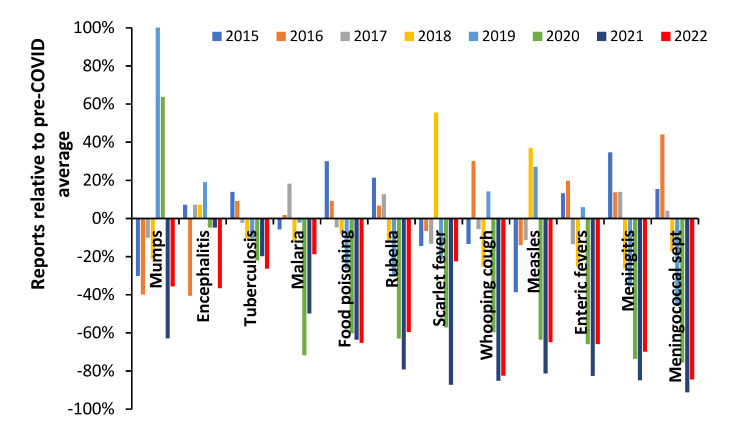
Notifiable disease statutory notifications (NOIDS) in England and Wales as a percentage difference relative to the average in the pre-COVID era 2015 to 2019, calendar years 2015 to 2022 [162]. Footnote: 2022 has been estimated from 2022 up to week 31 relative to 2021 at week 31 multiplied by the 2021 annual total. Sept = septicemia.

The behavior seen in influenza activity therefore seems to conform to pathogen interference by COVID-19 rather than to any major role from lockdown. Lockdown has been incorrectly attributed due to a lack of wider knowledge regarding pathogen interference. However, regarding other non-influenza pathogens non-vaccine epidemiological interventions during COVID-19 will have played a role in the reduction in person-to-person transmission [161]. An example is given in Figure 3 for notifiable infectious diseases in England and Wales in the years before and after COVID-19.

During 2020 in England there were approximately 175 days of lockdown and in 2021 approximately 90 days [160], hence the lower number of NOIDS in 2021 across many infectious diseases cannot be explained by relative days of lockdown. All COVID-19 restrictions were lifted in England on 24 February 2022. Data for 2022 include the Omicron outbreaks with limited measures to control spread other than by vaccination.

The trend for mumps includes a mumps outbreak during 2019 which continued into 2020. There is no evidence that the incidence of encephalitis was affected, which is consistent with its general non-transmissible nature. Tuberculosis (TB) incidence reached a peak in 2015 and declined subsequently. COVID-19 seemingly acted to reduce the downward trend in TB possibly by exacerbating existing TB infection [163]. Food poisoning is an interesting case since eating out in restaurants virtually ceased during lockdown, but consumption of take-away food increased [164]. The net effect was higher consumption of home cooked food and hence the observed reduction in food poisoning. During 2022 persons with COVID-19 should not be eating out due to self-isolation. Meningitis is a transmissible disease and showed a larger reduction than other conditions which looks to be mainly due to COVID-19 pathogen interference since all restrictions were removed in early 2022 [160], but new strains of COVID-19 were highly active. Meningococcal septicemia and whooping cough likewise appear highly susceptible to COVID-19 pathogen interference. Scarlet fever appears to have been a mix of lockdown and then secondary pathogen interference (perhaps less susceptible to the 2022 COVID-19 strains).

Hence, during the COVID-19 era, influenza was principally targeted by COVID-19 pathogen interference while other transmissible diseases showed a mix of pathogen interference and reduced transmission due to lockdowns and other public health measures.

### 5.7. Studies Implying Pathogen Interference in the Net Effects of Influenza Vaccination

Several studies suggest that influenza vaccination may be giving paradoxical outcomes against total population morbidity and mortality. Note that all studies about to be considered are for the total population and will therefore include the net effects of the benefits of influenza vaccination against subsequent influenza infection, counterbalanced by potential increased susceptibility to infection by other pathogens.

In the first study, levels of ILI (whole population or aged 65+) in 14 European countries were correlated against changes in vaccination rates for the whole population (range 0% to 33%) or in those aged 65+ (range 2% to 84%) [165]. Data were available for between 8 and 23 years for 12 different date ranges between 1991/92 and 2013/14. The average of the net effects of influenza vaccination against EWM as per Figure 2 [3] ranged from −1% for France (date range 2001/02 to 2011/12) to +0.9% for Slovakia (date range 2006/07 to 2012/13). Correlation of the apparent effects of influenza vaccination upon ILI or ARI against average vaccination effect for the years covered in each country (from Figure 2 [3]) gave a slight positive association for both the all-age and elderly ILI/ARI, i.e., in both cases an apparent negative effect of influenza vaccination was associated with an average disbenefit in those years from Figure 2 [3]. This merely reinforces the observation that the period covered by any study is critical to the conclusions regarding the potential roles for pathogen interference—as measured by ILI in this study.

In the next study, a Serfling–type seasonal model to define the baseline, excess winter mortality in the USA was followed during a period of rapidly expanding age 65+ vaccination rates (from 15% to 65%) between 1980 and 2001 [166]. The authors stated, “we could not correlate increasing vaccination coverage after 1980 with declining mortality rates in any age group”. A variety of methods were employed to discount possible confounding effects and the authors calculated that based on vaccine VE a negative trend was feasible, but not observed. These authors suggested that due to the high year–to–year volatility in influenza activity and EWM further international studies were warranted.

The third study looked at monthly age–banded all–cause mortality in Italy between 1980 and 2001 using a seasonal regression modelling approach. The authors concluded that “after the late 1980s, no decline in age–adjusted excess all-cause mortality was associated with increasing influenza vaccination distribution primarily targeted for the elderly” [167]. Note that this study is a multi-year average.

In the final study, a “difference in differences” approach was applied to winter deaths between 1996/97 to 2004/05. The resulting odds ratio was converted into a hypothetical VE. Strictly speaking this study was partly measuring the net effects on mortality where influenza vaccination was only associated with a small net benefit, although the confidence intervals overlapped no net effect [168].

It is therefore clear that the evidence has existed to suggest that the whole population effects of increased influenza vaccination may contain unanticipated outcomes.

The study of Sundaram et al. [169] over the six years 2004/05 to 2009/10 in Wisconsin, USA yielded no apparent effect of influenza vaccination on non-influenza viruses in children < 5 years and adults > 50 years. However, this study is an average over six years during which the average effect of influenza vaccination upon EWM was only −0.3% at 100% vaccination, from [3] and Figure 2. Hence, this study does not disprove that influenza vaccination alters the pathogen balance in individual years since it happened to occur over a period when the average net effect was close to zero [3].

## 6. Issues Relating to Influenza Vaccine Effectiveness

While both influenza serotypes: A and B contribute to seasonal outbreaks, only serotype A contributes to pandemics. Influenza viruses of A serotype are divided into serosubtypes based on the antigenic peculiarities of the two surface proteins: Hemagglutinin (HA) and Neuraminidase (NA). Hence, 18 hemagglutinin and 11 neuraminidase subtypes exist, with 198 potential subtype combinations of which 131 have been detected in humans [170]. A multitude of genetic variants called clades and sub-clades lie below the subtypes [170]. The timing of influenza outbreaks and their causative agents in terms of virus serotype(s) and serosubtype(s) or their mixture which vary considerably between countries each year. Current influenza vaccination technology is therefore akin to attempting to shoot an agile and fast-moving target, and this is reflected in highly variable vaccine effectiveness (VE) in each season [24].

Given the reality of pathogen interference, and the known confounding of VE estimates by pathogen interference [137], it is relevant to explore what exactly VE is measuring and if its calculation contains additional hidden assumptions.

### 6.1. Vaccine Effectiveness Estimates in the Real World of Multiple Pathogens

Influenza VE focusses on vaccination status (yes/no) and confirmed influenza infection (yes only). Symptomatic influenza infection only ranges from less than 4% to 12% of the population [91,114], hence, VE measurement is restricted to a very small proportion of the elderly population. The key question is what is happening in the other, far larger, proportion of the elderly population who have been vaccinated yet die over the winter. This is strongly implied by Figure 2—and emphasizes the importance of investigating all-cause mortality. It is also important to note that 32% of samples in Table 2 were negative for the 21 common pathogens, suggesting that other less–common pathogens were also involved in ARI [103], and presumably in further combinations of pathogen interference as per Table 1. Given the above it is highly unlikely that VE estimates are independent of pathogen interference.

For example, up to 5 of the common respiratory pathogens were detected in a single sample, and viral—bacterial co-detection was higher than viral—viral [103]. The persistent immune–modifying virus Cytomegalovirus (CMV) exerts its effects by stealthy immune manipulation rather than acute infection and infects many of the same lung cells as does influenza.

A study regarding co–infection between 13 common viral pathogens revealed that Adenovirus C had the highest co–infection rate while influenza B had the lowest. ADVC–rhinovirus, respiratory syncytial virus A–rhinovirus and RSVB–rhinovirus pairings occurred at significantly higher frequencies. Several pairings had fewer co–infections, namely, hMPV–PIV 3, hMPV–RSVA and RSVA–RSVB [120]. Hence, among those receiving an influenza vaccine, complex non-influenza infections will be prevalent—which is currently assumed to be unrelated to or influenced by influenza vaccination. As noted earlier, influenza vaccination status is rarely recorded in pathogen interference studies.

In a study between 1996 and 2005 the “net” VE was just 4.6% (CE 0.7% to 8.3%) [167]. The word “net” has been used to indicate that death due to the unintended consequences of influenza vaccination regarding pathogen interference would be included in the VE estimate. Note that the years 1996 to 2005 correspond to a period when the net effects of influenza vaccination yielded an average benefit of only 0.2% against EWM at a theoretical 100% vaccination—as per Figure 2 [3].

It goes without saying that different contexts for VE yield markedly different VE estimates, hence in the winter of 2009/10 in Scotland vaccination against Influenza A(H1N1) was 77% against influenza infection in a primary/ambulatory care context but only 20% for emergency hospital admission [170]. In the southern hemisphere, winter of 2013 in New Zealand VE in primary care was 76% compared to 34% for hospitalization [171,172].

To evaluate the possibility for cross–reactivity of vaccine–induced anti–influenza immunity (specific antibodies and immune effector T– and B–cells) with RSV, we performed a search using BLASTP online service [173]. Results showed up to 62% of amino acid sequence similarity at some selected regions. Detected sequence similarity represent a basis for cross–reactivity between acquired influenza immunity and RSV. It is clear that the interactions between influenza(s), influenza vaccination and RSV may be far more nuanced than appreciated.

### 6.2. What Does Vaccine Effectiveness Measure

In their comprehensive review Lang et al. [174] concluded “this review demonstrates that the achievement of an accurate assessment of vaccine benefits is still fraught with considerable methodological and epidemiological challenges”. A study over eight years concluded that the calculated benefits of influenza vaccination (VE) could be almost completely explained by selection bias [169]. The review of Thomas regarding VE in those aged 65+ concluded that the results were highly unreliable [175].

As pointed out above, one of the major limitations of assessing influenza VE is that VE is most often determined using a very small and specific sample from the total population. The most common form of VE is in an ambulatory care context (visits to a general practitioner or an emergency department). Due to the ambulatory/outpatient nature of this sampling method the elderly is vastly under-represented.

A study among Italian elderly aged 65+ concluded that from the 1980s there was no reduction in all-cause mortality associated with increasing influenza vaccination rates [168]. Such studies appear to contradict cohort studies claiming a 50% reduction in total winter mortality from influenza vaccination [176]. It was concluded that serious frailty-selection bias and the use of non-specific endpoints led to gross over-estimation of influenza vaccination benefits [176]. Other systematic reviews have reached the same conclusion [177]. Another review concluded that the net effect of influenza vaccination was unlikely to give appreciable changes in the financial and capacity risks experienced in health care during the winter [100]—even though influenza vaccination is reducing deaths from influenza per se.

Another study concluded that different types of study, i.e., cohort, case–control, were subject to a seeming high bias to overestimation of the net benefits of influenza vaccination [178]. Is this disquiet with VE symptomatic of deeper issues?

### 6.3. Pathogen Interference and Variation in VE

**Figure 4:**
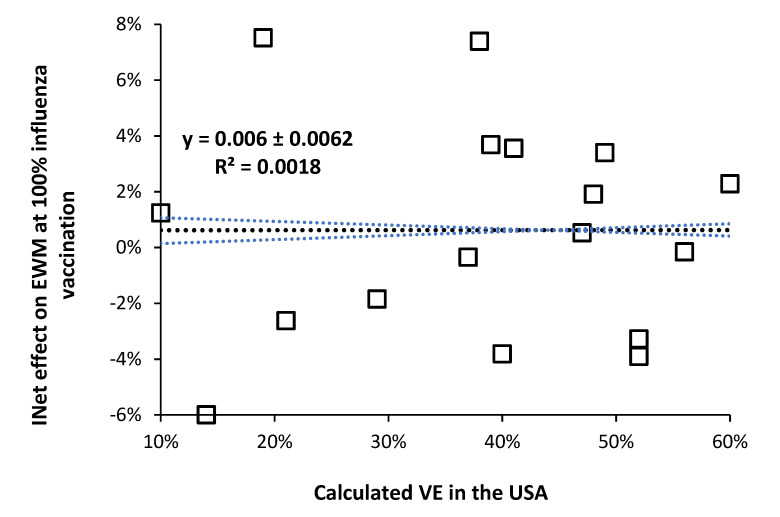
No relationship between calculated VE (under the limiting assumption for no role of pathogen interference) and the effect of 100% influenza vaccination upon international all-cause excess winter mortality, from [3].

As shown in Figure 4, when the data from Figure 2 [3] are plotted against the calculated VE in the USA for the same years [6], there is no correlation between the two (R-squared = 0.0018). Hence, whatever is altering the net effects of influenza vaccination upon all-cause excess winter mortality is independent of whatever may be involved in the calculation of VE (under the limiting assumption of no role for pathogen interference).

This is an interesting observation because during a high VE year the ensuing diminution of influenza activity might be expected to cause a shift to other respiratory virus infection in the vaccinated. However, there is no evidence that high VE regulates the incidence of influenza—which occurs via meteorological factors (mainly temperature) [92,93,94,95,96,97,98], and pathogen interference. Clearly the mechanism for the effect of influenza vaccination lies elsewhere or the calculated VE is being subverted by the different levels of pathogen interference each winter.

This is perhaps a relevant point to raise yet another hidden flaw in VE which arises from the study of McLean et al. [179], namely that the measured VE is highly single-year-of-age dependent. By extrapolation the calculated age 65+ value of VE which is universally reported becomes highly dependent on the method of age standardization, i.e., do we use a standard population age distribution (world population, country population, etc.), or the current years age distribution to better reflect the impact of the ageing population, or do we correct for the age profile of deaths, or the age profile of influenza admissions, etc. Each method will give different answers for the calculated age 65+ VE. This is important because it is relevant to what the current calculation method is intrinsically measuring, and if it is of fundamental relevance. Indeed, is the method of McLean et al. [179] to show VE by year-of-age of more intrinsic relevance?

**Figure 5:**
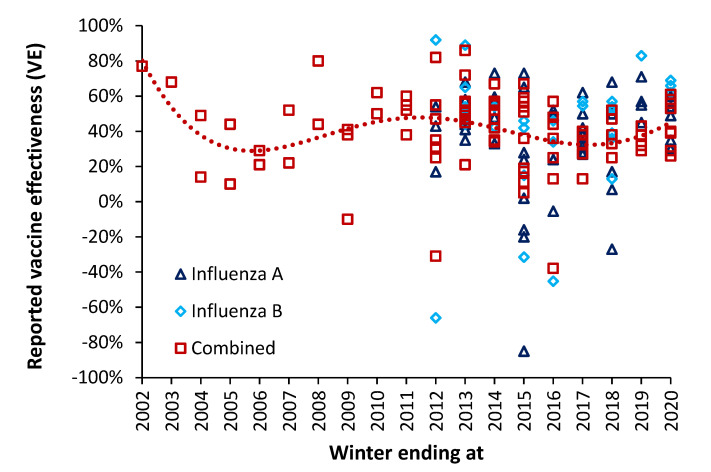
Longitudinal behavior of international vaccine effectiveness (VE) studies. Data come from a random search using Google Scholar covering different countries, and both interim and final year estimates [24,123,128,129,130,131,132,133,172,173,180,181,182,183,184,185,186,187,188,189,190,191,192,193,194,195,196,197,198,199,200,201,202,203,204,205,206,207,208,209,210,211,212,213,214,215,216,217,218,219].

However, given the known ability of winter pathogens to either enhance or diminish influenza infection it is suggested that pathogen interference should contribute to the observed high volatility in VE [24] both between years and within the same year but between countries. While it is widely recognized that VE varies considerably between years [24], it is perhaps less widely recognized that VE varies between counties within the same year—see Figure 5. It is suggested that the wide variation in VE between counties in the same year is also an outworking of variable levels of pathogens between countries and years.

It is acknowledged that VE estimates have wide confidence intervals and that new clades are emerging constantly in different locations, however, there appears to be additional factors involved in the observed wide variation. Confirmation of this would require wider international reporting by Public Health agencies of effective VE regarding the non–influenza group and larger samples than usually employed, and over a prolonged time.

Given the ability of pathogen interference to modulate influenza incidence, and of influenza vaccination to alter the pathogen balance, are there wider studies supporting the notion of an unintended effect of influence vaccination against winter mortality.

### 6.4. Negative Vaccine Effectiveness and Pathogen Interference

Negative vaccine effectiveness arises when the antigen mix in the influenza vaccine shows considerable antigenic mismatch against the actual circulating strains [2,3,220]. It implies that the outcome in the vaccinated is worse that for the unvaccinated. Such worse outcomes can arise from antigenic priming and/or heterologous immunity. Negative VE is moderately common and was first documented during the 2008 Swine flu pandemic—as discussed in Hearn [221]—although it had probably occurred prior to this but lacked the confirmation via PCR-based test negative studies which began around 2003.

Negative VE against certain strains is reasonably frequent (Figure 5) and seemed prevalent in the 2014/15 season in certain countries when excess winter mortality (EWM) was particularly high. As examples of negative VE, a study during the 2010/11 season showed VE variation associated with influenza serotype, A(H3N2) 10%, A(H1N1) 26% and B 48%. However, for individuals vaccinated in the previous year VE was negative, A(H3N2) −34%, A(H1N1) −6% and B −166% [222]. In Israel, during the 2016/17 season VE over the age of 65 went negative (−116%) [180]. In 2014/15 VE for the elderly (aged 65+) was particularly poor and in Italy was 72% for influenza B, but only 1% for A(H1N1), and (negative) −69% for A(H3N2) [181].

The interaction between negative vaccine effectiveness and pathogen interference remains to be investigated.

### 6.5. Peculiar Longitudinal Behavior of International Vaccine Effectiveness (VE)

The output from a previous study [2] suggests that the longitudinal calculated value of VE should be exhibiting undulating behavior, and this is illustrated in Figure 5 which takes a random sample of international VE estimates over the winters 2001/02 through to 2019/20 [6,123,128,129,130,131,132,133,172,173,180,181,182,183,184,185,186,187,188,189,190,191,192,193,194,195,196,197,198,199,200,201,202,203,204,205,206,207,208,209,210,211,212,213,214,215,216,217,218,219,222].

The implications of this review are that VE between countries should show high scatter over and above that due to the sample-size related uncertainty surrounding each VE estimate (not shown). The second point is that the international time-trend of VE is showing undulations, and that long-term undulations in EWM were shown to be a common feature of all-cause EWM in a previous study [2]. As has been pointed out by others the calculation of VE makes the key assumption that VE is not influenced by hidden or ‘emergent’ factors [137,169], which this review has demonstrated do exist.

### 6.6. Roles for Age and Nearness-to-Death

The issue of age is highly relevant. All-cause excess winter mortality increases rapidly with age especially above the age of 65 [1,216]. In the UK in 2018 the most frequent age to die was 83 in males and 88 in females. Some 50% of all deaths occur above the age of 79 in males and 84 in females [216]. Most common age to die has been above age 80 since 2000 [216], hence the traditional age 65+ to measure VE in the elderly is no longer representative.

It is widely considered that most winter and influenza deaths occur for age 65+ [91,217]. However, influenza vaccination effectiveness (VE) is generally considered to be highest in children and lowest for age 65+ [6]. VE for the elderly is surprisingly mediocre and, in the USA, saw an 18–year maximum of 60% in 2010/11. The median VE during 18–years was only 40% with an interquartile range (IQR) of 23% to 49% [6]. Hence, in an average year only 40% of the elderly benefit from influenza vaccination, leaving potential for unanticipated outcomes. However, such broad-brush statements conceal a world of far greater complexity.

As mentioned above, in one of the few studies using age as a continuous variable the dependance of VE on age was shown to be somewhat more complex that commonly appreciated. During the 2012/13 influenza season in the USA, VE against Influenza A (H3N2) had a maximum of 60% at age 1, fell to a minimum of around 18% around age 23 (those born around 1990), rose to another maximum of around 40% at age 48 (those born around 1955) and then declined with age to around 16% at age 76 (those born around 1937) [179]. Extrapolation of the data gave VE of 0% around age 90 (year of birth 1923). Presumably VE could go negative above age 90. This study also made the important observation that VE for influenza B was higher at all ages, reached an earlier peak around age 43 and then showed only gradual decline with age with no suggestion of reaching negative VE. The use of an age 65+ band (universally used in VE estimate studies) for VE is misleading since the study of McLean et al. [179] established the principle of age-dependence.

**Figure 6:**
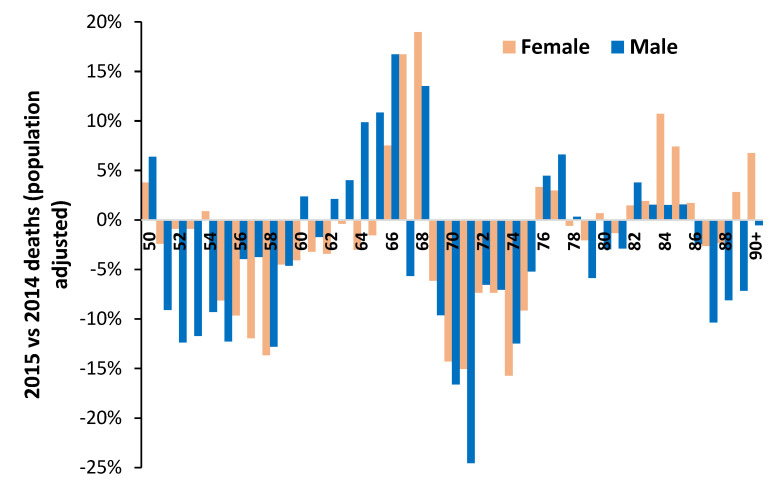
Effect of single-year-of-age on total deaths (population adjusted) in 2015 versus 2014 in England and Wales [216,218]. Footnote: Population data are only available for age 90+. Age 50 was chosen as the cut-off since there are more than 1 000 deaths per year beyond this point and Poisson variation is minimized. Above age 83 one standard deviation (STDEV) of Poisson variation is less than ±1%. In addition, there was a statistically significant (+7.2 STDEV) 18% increase in deaths of male infants (first year of life).

Regarding the issue of age dependence Figure 6 illustrates the potential for further hidden patterns. Figure 6 is for single-year-of-age all-cause mortality in England and Wales in 2015 compared to 2014. This combination was chosen due to a very large spike in international total winter deaths in early 2015 [219]. This was especially the case in England and Wales [223], where the winter of 2013/14 was innocuous, and this allows the detailed comparison of deaths at single-year-of-age for the two calendar years. Public Health England initially estimated only 3% VE (early season estimate) but later revised this estimate to 34% for the final season estimate [223]. A curious anomaly given that levels of ILI were surprisingly low during the early spike in deaths. Recall that infection precedes death and deaths usually peak around one month later [54]. It has been suggested that influenza was interacting with an outbreak of a second pathogen [224]. However, as can be seen in Figure 6 (after adjustment for population changes by age between the two years) the resulting profile is highly age and gender dependent. This method works because the majority of excess winter mortality occurs in January to March, at the start of the calendar year.

**Figure A1:**
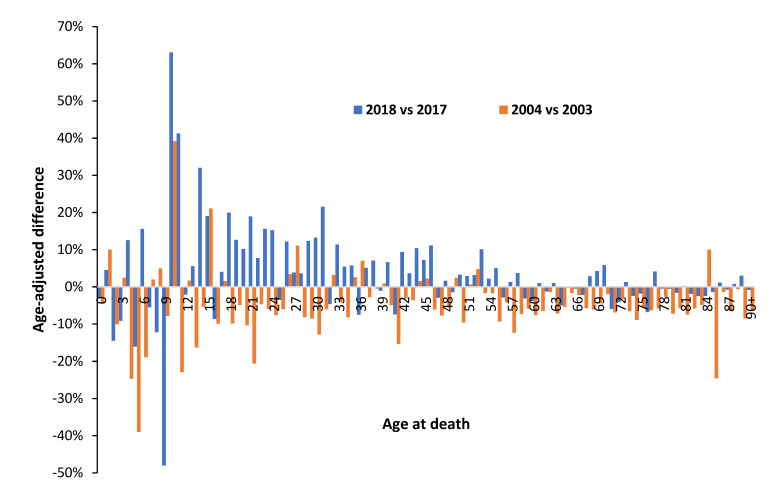
The age-specificity of population-adjusted male deaths in 2018 versus 2017 (high mortality) and 2004 versus 2003 (low mortality). Data sources as per Figure 6.

The winter of 2017/18 likewise saw high international mortality associated with influenza vaccination [3], and in England and Wales this was reflected in high deaths in 2018 versus 2017 [216]. The winter of 2003/04 was, however, a low mortality winter. Figure A1 in the Appendix A presents a similar chart to that in Figure 6 for population-adjusted all-age male deaths in 2018 versus 2017 and 2004 versus 2003 [216,218]. For the high mortality winter 2017/18 males aged 10 (born in 2008) to 45 are especially affected, although with single-year-of age patterns. Male children aged 9 (born 2009) show specific low mortality. In contrast the low mortality 2003/04 winter comparison show far less single-year-of age variation. The single-year-of-age patterns are attempting to communicate something of importance.

Note the profound effect of gender in Figure 6. Gender has an enormous effect on all aspects of healthcare [99,100] but is a completely neglected area in VE studies. Next note that both the study of McLean et al. [179] and Figure 6 and Figure A1 completely contradict the widely held view that immune function declines with age, called immunosenescence. Indeed, primary roles for immunosenescence upon vaccine efficiency in the elderly have been questioned [225]—demonstrating it is not age per se that regulates immunosenescence. How does immunosenescence explain the minimum VE at age 23 seen in the study of McLean et al. [179], or the fewer male deaths above age 87 in 2015 in Figure 6? Indeed, in the study of McLean et al. [179] age was represented as a restricted cubic spline function with 5 knots based on percentiles, i.e., the single-year-of-age profiles in Figure 6 would have been largely smoothed out by the 5 knots cubic spline method. A presumed infectious outbreak in early 2012 led to a different single-year-of-age pattern between deaths in 2011 and 2012 [226]—hence such patterns are perhaps more common than realized and are seemingly of great significance regarding the potential causes.

The most common measurements of VE use only five broad age bands, namely, age 0–8, 9–17, 18–49, 50–64, 65+ [24]. Due to the outpatient nature of most VE estimates, age 65+ is vastly underrepresented with just 1 301 persons aged 65+ out of 10,012 (13%) in the US CDC 1018/19 VE estimates which are based on emergency department (ED) attendances [24]. Some 33% were aged 18–49 [24]. Such broad age bands are completely obscuring the real mechanistic detail. Issues of selection bias are probably more prominent in the USA due to the vagaries of medical insurance and ED usage.

**Figure A2:**
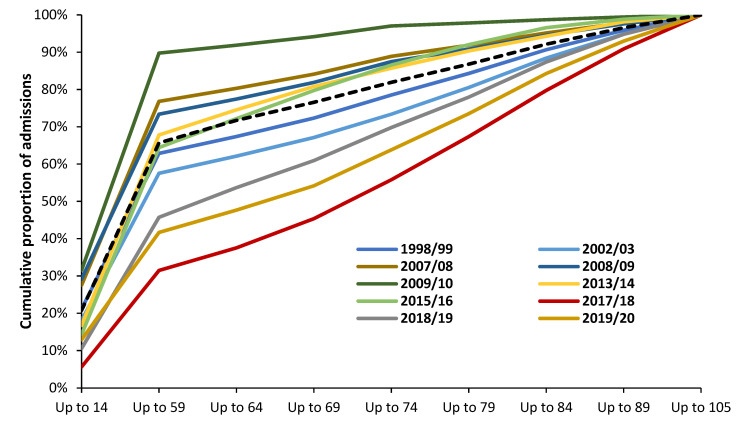
Cumulative proportion of influenza (ICD J10 and J11) admissions for different age groups to English NHS hospitals for various financial years, 1998/99 to 2019/20. Data are from NHS Digital, Hospital Admitted Patient Care Activity—NHS Digital (https://digital.nhs.uk/data-and-information/publications/statistical/hospital-admitted-patient-care-activity accessed on 1 September 2022).

**Figure A3:**
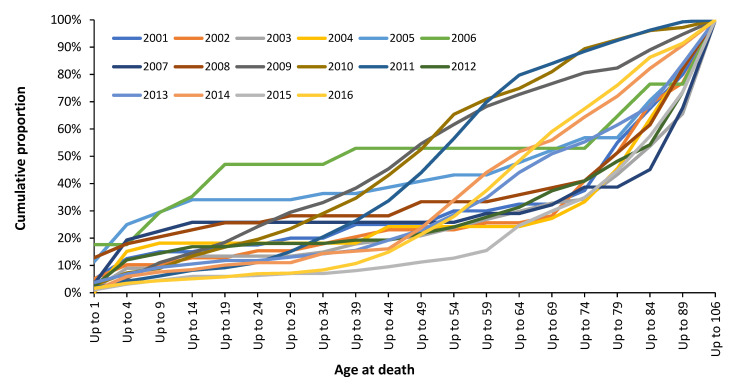
Cumulative proportion of ‘caused by’ influenza deaths in England and Wales for different age groups over the period 2001 to 2016. Before 2009 coding of deaths to influenza is very low. Data are from the Office for National Statistics: Number of deaths where influenza was the underlying cause of death or was mentioned on the death certificate, by five-year age group, England and Wales, 2001 to 2016—Office for National Statistics (ons.gov.uk) (https://www.ons.gov.uk/peoplepopulationandcommunity/healthandsocialcare/causesofdeath/adhocs/007849numberofdeathswhereinfluenzawastheunderlyingcauseofdeathorwasmentionedonthedeathcertificatebyfiveyearagegroupenglandandwales2001to2016, accessed on 1 September 2022).

Indeed, the profound hidden assumption in all VE estimates is that the age profile of both genuine ‘caused by’ influenza hospital admissions or deaths is highly dependent on the influenza season, as was implied for Figure 6. This is illustrated in Figure A2 (hospital admissions) and Figure A3 (deaths). Note that both these figures are for influenza-only confirmed (caused by) admissions or deaths. While single year of age data are not available the vast variation in the age profile between years is clear.

Somewhat concerningly, the broad-brush statement that most influenza (meaning influenza plus pneumonia) deaths occur above age 65 is entirely unsupported by Figure A2 and Figure A3. In Figure A1 in 2009/10 only 8% all-age influenza admissions occur for age 65+, while this proportion is 62% for 2017/18. The median over 22 years is only 28% of total influenza admissions over age 65+. The same applies in Figure A3 where only 20% of influenza deaths occur above age 65+ in 2011 (as a proxy for the 2010/11 season), while 75% of influenza deaths occur above age 65+ in 2015 (as a proxy for the 2014/15 season). This predominance of elderly deaths for 2014/15 is confirmed by Figure 6—although with single-year-of-age specificity. Recall that in Figure 6 the most frequent deaths are for those aged in the mid 80’s and this skews the impact of the percentage changes for each age.

**Figure A4:**
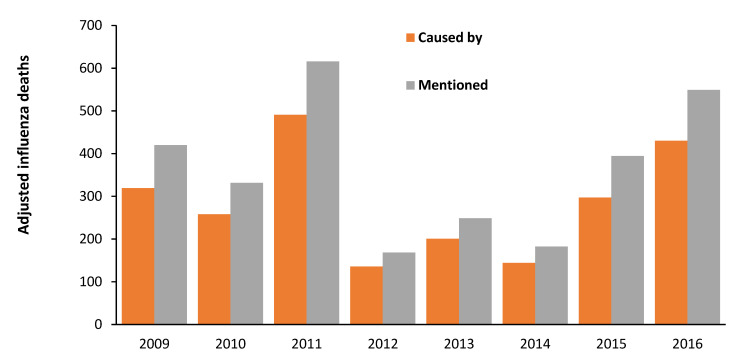
Influenza deaths in England and Wales for the years 2009 to 2016 where the death is directly due to (caused by) influenza or where influenza is mentioned in any place in the death certificate. Data are from Figure A3. Deaths have been corrected for underlying growth of +13.1 extra deaths per year for ‘caused by’ or +16.6 for influenza mentioned deaths. Underlying growth arises from increasing population and changing age profiles.

As was mentioned above there was a very large increase in deaths in 2015 (as per Figure 6), however, Figure A4 shows that this was only partly due to influenza—which reinforces the proposal that a second pathogen was also involved [100,224]. Additionally, note from Figure A4 that ‘influenza’ deaths are surprisingly low. Despite the widespread availability of PCR tests for influenza in recent times, the study of Doshi [227] also noted a surprising low number of confirmed influenza deaths in the USA. Hence, the reason that ‘estimated’ influenza deaths include a proportion of other causes—although such estimates are open to hidden assumptions [2]. For example, the age profile for the proportion of other diagnoses must match that shown by influenza (as in Figure A1, Figure A2, Figure A3 and Figure A4). If it does not, then non-influenza deaths are being incorrectly attributed to influenza. These assumptions around age-standardization in influenza VE probably contribute toward the lack of correlation shown in Figure 4. The above sections strongly suggest that the influenza narrative may contain hidden flaws among which include inflated estimates of influenza deaths [2]. This is vastly important because it could imply that it is the intermittent consequences of influenza vaccination per se which is acting via non-specific innate immune effects.

Section 3.5 regarding antigenic original sin/immune priming offers the greatest insight into such complex patterns. The original study by Francis [228] specifically noted that antigenic original sin created unique age profiles which depended on the antigenic distance between the first influenza strain a person had encountered and the most recent infection. In essence, vaccination attempts to override the acquired immune patterns and responses and will create additional complex patterns somewhat resembling phase interference—hence the patterns in Figure 6 and the study of McLean et al. [220].

The age 68 cohort in Figure 6 were born in 1947 and would have been first exposed to influenza strains circulating at that time. High deaths indicate a large antigenic gap between the 1947 and 2015 strains—precipitating negative vaccine effectiveness and the high deaths, etc.

How such effects may interact with pathogen interference remains to be explored. The issue of chronological age versus nearness to death can now be addressed.

Up to the present chronological age has been assumed to describe most medical phenomena. In the seminal paper of Nicholl [229] age is shown to involve the constant risk fallacy, i.e., age is used as a crude proxy for nearness-to-death. Almost all VE studies to date have used diagnosis to (poorly) circumvent the nearness-to-death or time-to-death effect [230]. However, it has been noted that during the last two years of life there is a progressive increase in physical and cognitive frailty [231,232,233]. There are different frailty trajectories in different risk groups [231,232,233], and hospital admissions dramatically increase in the last year of life [234]. A composite score based on common blood biochemistry results only increased slowly with age but underwent a dramatic shift during the last months of life [235].

Respiratory infections are very common in nursing home residents [236]. To a great degree such persons are waiting for any event capable of precipitating final demise. In years when influenza is higher, influenza simply becomes the event which precipitates final demise—or a non-influenza pathogen in the years when influenza vaccination seems to precipitate higher pathogen interference. One study showed that adjustment for functional status (as a proxy for nearness to death) reduced the apparent VE by 20% [237]—implying that current methods are over-estimating VE, and probably the incidence of negative VE. Once again, nearness to death remains unexplored territory in terms of both VE and pathogen interference. Indeed, rapid functional decline in the last six months of life could imply that vaccination becomes ineffective during this period.

Finally, the issue of why influenza vaccination should commence at age 65 requires consideration. While it may be true that all-cause excess winter mortality shows a small increase at age 65—those nearest to death?—most adults of this age are healthy. We propose that immunosenescence only occurs very slowly with age per se but shows rapid decline with nearness to death. The nearness to death effect then contaminates studies involving age generating the illusion that immunosenescence increases with age [229].

Two very large regression discontinuity design studies regarding influenza vaccination at age 65 both demonstrated a very small or no effect. In the first, a study in England involving 170 million hospital episodes and 7.6 million deaths between 2000 to 2014 looked for changes in the hospital admission rate and mortality around the age 65 boundary where the population is widely vaccinated against influenza [179]. The authors concluded that “no evidence indicated that vaccination reduced hospitalizations or mortality among elderly persons” [179]. This study occurred over a period (2000/01 to 2013/14) where the average net effect of influenza against all-cause EWM was a 1.1% reduction in EWM from Figure 2 [3].

A similar regression discontinuity design study in the Netherlands at the 65–year age boundary found that influenza vaccination “had a small to negligible effect on hospitalizations and influenza/pneumonia deaths at age 65” [238]. This study occurred over a period (1997/98 to 2007/08) where the average net effect of influenza against all-cause EWM was a 0.9% reduction in EWM from Figure 2 [3].

Hence, both studies occurred during periods when there should have been a very small reduction in winter deaths. However, the confidence intervals from these two studies overlap the slight reduction in deaths predicted from the previous study which covers all-age mortality [3].

Had these studies been conducted at different times the results could be markedly different. For example, a study conducted between 1986/87 to 1995/96 would have been during a period of an average net benefit of a 2.9% reduction in EWM from influenza vaccination, while a study conducted between 2008/09 to 2017/18 would have had an average net disbenefit of a 1.9% increase in EWM as per Figure 2 [3]. In both cases the high season to season variation would have led to the large confidence interval observed in both the above studies.

The issue of the 65–year boundary was hinted at in Section 6.2 where it was noted that while children and the elderly showed evidence for pathogen interference the results were less clear for working age adults. Age 65 is at the upper edge of working age and the null effect of vaccination can indicate either of two things.

1. Pathogen interference is operating such that any benefits of influenza vaccination are counterbalanced by pathogen interference.
2. Age 65 is too young to commence influenza vaccination.

Regarding #2 it should be noted that in the UK the most common age to die is around 85 years [216].

The results of this and earlier studies [2,3] seem to indicate that influenza vaccination may be having unintended consequences and should be targeted to high-risk individuals (as in past years) rather than blindly applied based on an arbitrary 65+ age boundary—which may have been relevant decades ago when life expectancy was far lower.

The above may seem somewhat abstract, however, it is important to understand how age per se and nearness-to-death are interacting with influenza vaccination, VE, and pathogen interference. VE estimates based on ‘outpatient’ type attendances for ILI are assumed to involve very few in the nearness-to-death group, while winter mortality is almost exclusively to do with this group.

## 7. Roles for Temperature and Pollution

There are complex interactions between air pollution (PM10), temperature and influenza activity (measured as ILI) on all–cause, respiratory, and cardiovascular mortality [238]. Each of these variables operates both alone (PM10 mainly affects cardiovascular and influenza mainly respiratory) and in combination with additional specific interactions between influenza and PM10 for cardiovascular mortality and between influenza and temperature upon all-cause mortality [239]. The relationships are complex and are likely to contribute both to pathogen interference and the variability in EWM. Note that influenza activity was approximated by ILI which has been proposed to be more a measure of pathogen interference that influenza activity per se. It has been suggested that respiratory viruses adapt their thermal sensitivity to local conditions, hence influenza outbreaks in the tropics [240]—implying a range of influenza variants with latitude.

**Figure 7:**
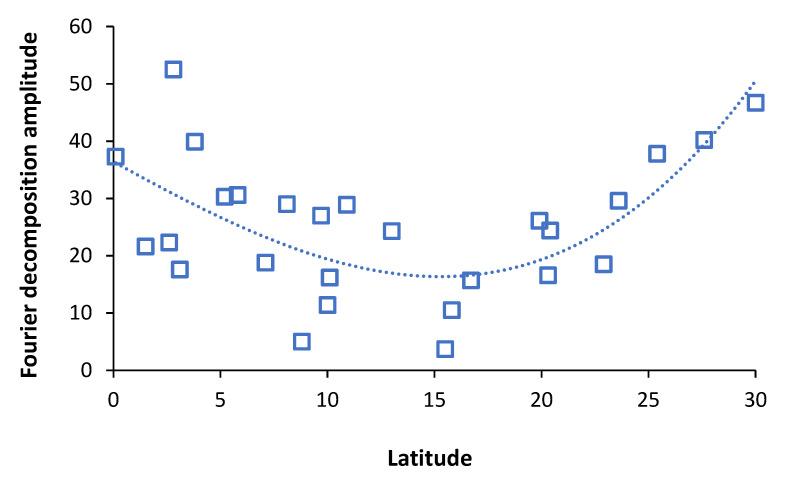
Role of the latitude of Brazilian states on the average amplitude of winter influenza (pneumonia + influenza) deaths, 1979–2001. Northern/Southern hemisphere latitudes all shown as a positive number. Adapted from Alonso et al. [241].

Figure 7 gives an interesting view of the role of latitude—as a proxy for temperature and other meteorological variables—on the average amplitude of influenza + pneumonia deaths in various Brazilian states [241].

Note that midway between the equator and the poles is 45°. Interestingly microbial species diversity peaks around latitude 45 [30]. Latitude is merely a proxy for local weather patterns, hence the scatter around the trend line in Figure 7. It is possible that the amplitude reaches a maximum around 45°and then declines closer to the poles [1]. The Brazilian study noted that influenza/pneumonia outbreaks originated at the equator. The minimum around latitude ± 15° remains to be explained as does the higher scatter around the trend line between ±15° and the equator. Additionally, note that the role of altitude [3] also seems to be important.

The distribution of pathogens and infectious diseases per se is known to be highly latitude dependent being generally more diverse near the equator [242]. By latitude 60° N species diversity has significantly declined [30]. There have not been any specific studies investigating the role of latitude on the relative prevalence of influenza and other respiratory pathogens or how this may affect pathogen interference. However, the expression of immune genes shows seasonal variation [243,244,245] as does the expression of miRNAs [243]—which are implicated in pathogen interference. Figure 7 therefore most likely reflects the nuances of pathogen interference.

Since most of the excess winter deaths occur in world cities—which are also the most polluted—the role of pollution via lung inflammation and potential disruption of the lung microbiome [246] remains to be explored with respect to pathogen interference and the net effects of influenza vaccination.

## 8. Comments Specific to Respiratory Syncytial Virus (RSV) and Pneumonia

### 8.1. Respiratory Syncytial Virus (RSV) and Attributed “Influenza” Mortality in the Elderly

Respiratory Syncytial Virus (RSV) causes as much respiratory–only mortality in the elderly as influenza [247,248,249,250], however, it is commonly misdiagnosed as “influenza” [156,247,248,249,250]. More severe presentations of RSV occur in the immunocompromised, cardiopulmonary disease and old age [247,248,249,250]. Combined sputum and nasopharyngeal swab are required to increase detection [247,248,249,250]. The proportion of deaths due to RSV depends on age and influenza season. Over 8 seasons for combined influenza + RSV at age 65–84 influenza accounted for an average of 60% of combined deaths range (22% to 75%), while for age ≥85 average 57% (range 30% to 68%) [249]. Hence, a subtle shift with age to higher RSV deaths. In addition, parainfluenza causes appreciable elderly deaths, and in those aged ≥85 for every 100 RSV deaths there are an average of 57 (range 34 to 77) additional parainfluenza deaths [249]. These can also be misdiagnosed as “influenza”. This is all in the context of an earlier study which showed that mortality directly due to pandemic influenza has almost certainly been over estimated—with the notable exception of the Spanish flu pandemic [2]. Note from Table 2 the different interactions between RSV A and B and influenza A and B.

An important study in the state of San Luis Potosí in Mexico between 2003 to 2009 (latitude 23 N, equivalent to Egypt, Saudi Arabia, India (Gujarat), Bangladesh, and in the Southern hemisphere to South Africa, Northern Territory Australia, Sao Paulo Brazil) showed that in the relative absence of influenza then RSV predominates. Influenza epidemics were less frequent than RSV, and that RSV was seen as the competitively dominant virus. Influenza had a long–term periodicity of around 128 weeks (approx. 3 years) while RSV had a 6–month periodicity [151]. They estimate that the reproduction number (R0) for influenza ranged from 0.6 to 1.58 while that for RSV ranged from 0.6 to 2.75. Suppression of influenza by vaccination has the real possibility of increasing RSV mortality to a greater extent than if influenza had been left in place. This effect may be latitude dependent and may partly contribute to the higher values of excess winter mortality at middle latitudes [1].

### 8.2. Interaction between Influenza and Pneumonia

It has been recognized for many years that bacterial pneumonia generally rises with influenza, and that it is the secondary pneumonia that then causes death [251]. The Spanish flu pandemic was especially lethal due to the promotion of bacterial pneumonia [252]. The influenza infection causes lung epithelial damage which then leads to a cascade of events precipitating bacterial infection [253]. It has been proposed that plasminogen-activating streptococci and staphylococci facilitate viral replication and pathogenicity of plasmin-sensitive influenza virus strains by amplification of the plasminogen/plasmin system [254]. One study estimated that influenza infection increased the risk of pneumonia by 100-fold, however, only in instances where bacterial infection lags viral infection by 5–7 days is there increased susceptibility [255]. It has been further proposed that complex interactions within the respiratory and gastrointestinal microbiota are also involved [256].

Influenza-induced pneumonia, which is a special case of pathogen interference, is therefore limited to a specific set of time-dependent cases. Pneumonia per se is therefore a gross over-estimate of influenza-induced deaths, the suspicion being that the proportion directly attributable to influenza may be lower than that included in current estimates. Hence, the suggestion in previous studies that real number of influenza deaths are being over-estimated [2,3] via pathogen interference.

## 9. System Complexity and the Unanticipated Effects of Influenza Vaccination

### 9.1. The Immunology of Why Vaccines Work

Firstly, it must be acknowledged that vaccination in general has been a remarkable success and has saved millions of lives [257]. However, most vaccines were developed empirically [258], and our knowledge of why vaccines work (from a whole system perspective) is a developing science [259,260,261,262]. Given the fact that influenza operates as part of a complex web of pathogen interference, the immunology of influenza vaccination may be more complex than anticipated. The phenomena of immune interference may also be involved by interfering with T-cell priming [263,264]. It is the ability of the human body to maintain the balance between pro- and anti-inflammatory forces, in the face of multiple persistent and transient infections which determines the ultimate pathogenicity of the next arriving infection [262], as per heterologous immunity in Section 3.4. Up to the present it has been assumed that influenza vaccination does not interfere with this balance.

### 9.2. Possible Role of Asymptomatic Infections

The role of human immune diversity cannot be overemphasized [70,71,72,265,266,267]. Asymptomatic infection is another example of human immune diversity. Asymptomatic infection by viruses is common [268], somewhere between 18% to 75% for COVID-19 infections, depending on world region [269], higher than 70% for 16 common viruses, 65% to 97% for common viruses in another study [270]. Below 50% for influenza and human metapneumovirus [267]. Asymptomatic rates for influenza were significantly higher for school children, ranging from 56% to 78% [271].

Live attenuated vaccines cause a type of asymptomatic infection. Asymptomatic infection is a result of specific peculiarities of a pathogen and/or host immunity. Influenza virus selection in natural or experimental conditions can result in the occurrence of viral subpopulation prone to cause long lasting asymptomatic infection [272,273].

Percentage of asymptomatic cases usually increases at the end of epidemics and especially in inter-epidemic seasons. These asymptomatic infectious processes represent the space for influenza population persistence, evolution, and adaptation in the inter-epidemic season, resulting in gradual selection of new virus strains with potential for epidemic spread in human (or avian, animal) population [274]. Specific molecular–biological, genetic, and biophysical features of influenza strain variants that correlate with their ability to form asymptomatic and persistent infection have been described [272,273,274,275,276].

Such hidden circulation of influenza viruses in human population in inter-epidemic period (seasons) may cause minor, region-specific modifications of the human population immunity. Hence, one reason for the strange local authority trends seen in Figure 1. At the beginning of a new influenza epidemics the frequency of both asymptomatic and sporadic symptomatic cases starts increasing. The role of human immune diversity in forming of asymptomatic infection cannot be overemphasized. For example, immunological hypo-reactivity can lead to establishment of chronic asymptomatic infection [277]. Role of pathogen interference and/or the respiratory microbiota in establishment of asymptomatic infection remains an unexplored area.

Thus, vaccination with WHO-approved influenza vaccines can elicit various immune responses in different people/locations depending on their immune status and possible asymptomatic infection at the time of vaccination.

### 9.3. Influenza Vaccination and HIV/AIDS

At this point the question must be addressed as to whether studies exist which show that influenza vaccination can alter the pathogenicity of another pathogen. A study demonstrated that HIV replication was increased during the 30 days following influenza vaccination using the 1993/94 season vaccine [278]. However, this response was highly individual specific—an issue we have repeatedly emphasized. In addition, it is unknown if this response changes with different seasonal vaccines and/or is influenced by adjuvants. It is assumed that the same stimulatory response may hold for other persistent and winter pathogens.

### 9.4. Unanticipated Effects on All-Cause Mortality by Other Vaccines

Other vaccines (BCG, polio, measles) have been shown to have unanticipated non-specific immune effects resulting in improved all-cause mortality over-and-above that expected from the specific disease targeted by the vaccine [279,280,281]. However, BCG vaccination increases the detrimental effects of subsequent malaria infection [280]. This is the equivalent to heterologous immunity in Section 3.4. It should be noted that these non-specific effects are gender specific [281].

Note that the antigenic composition of the other vaccines is fixed while that of influenza vaccines is variable. Hence, the question raised above, i.e., would a different seasonal vaccine lead to a different outcome in HIV replication [278].

### 9.5. Variable Responses to Influenza Vaccination

The molecular and gene signatures invoked by influenza vaccination over five seasons (2010/11 to 2014/15) showed high variation between individuals and between the young and the elderly [282]. Models explaining vaccine responses in the young did not apply to the elderly.

Another study showed that antibody responses (influenza seasons 2007/08 to 2011/12) correlated with age, although with high individual and seasonal variation [283]. Neural network analysis of gene transcriptional responses revealed some common patterns. Different antibody patterns will imply different patterns of miRNAs.

### 9.6. Pathogen Subversion of Antigen Presentation

Adenoviruses, Chlamydia trachomatis and many other virus and bacterial pathogens developed mechanisms for immune evasion. These “anti-immunological” mechanisms often are non-specific, cause disruption of immunity-related molecular pathways, and systemic inhibition of antigen presentation resulting in general inhibition of immunological responsiveness. For example, some pathogens can inhibit intracellular transport of the major histocompatibility complex (MHC) molecules or directly bind to them disrupting antigen presentation [284]. Therefore, persons infected with adenovirus, Chlamydia, Cytomegalovirus, Toxoplasmosis, rickettsia, or any other immune-subversion pathogen(s) would develop much weaker protection reaction upon vaccination in comparison with uninfected individuals. In such cases individual vaccine dose correction or use of specific adjuvants could be necessary. Thus, to achieve the anticipated effect from vaccination in each vaccinee, preliminary assessment of the candidates’ immune system and some virological/bacteriological tests are desirable. This remains a neglected area of pathogen interference and vaccination research.

### 9.7. Roles for Transcriptional Signatures and Small Noncoding RNAs (ncRNA) in Evaluation of the VE

Up to the present, the efficacy of influenza (and other) vaccination has been attributed largely to antibody production [285]. Thus, modern approaches to investigation, estimation and correction of virus–host interactions and antivirus (anti-infectious) reactions are predominantly protein—based (antigens, antibodies, cytokines) with some exceptions like diagnostic PCR and modern RNA vaccines for SARS-CoV-2 prophylaxis. However, this response is highly individual specific [286].

Meanwhile, scientific progress provides new opportunities for understanding and evaluation of the host–pathogen interaction, including investigation of vaccination mechanisms and vaccine efficacy. Study of transcriptional signatures and small noncoding RNAs (ncRNA) proved to be useful in this context [282,283,287]. It was shown that ncRNAs play an essential role during influenza infection. Hence, an alternative to the current global trend mentioned above is an RNA-based (including ncRNA-based) approach to disease prophylaxis, treatment and diagnosis. Investigation of the regulatory role of small virus RNAs (svRNAs) provide new options for diagnosis and therapy of infectious diseases. svRNA triggers the viral switch from transcription to replication through interactions with the viral polymerase machinery [288]. With respect to this possibility, it has been shown that:

- Pre-vaccination transcriptional signatures that were associated with antibody responses revealed numerous new types of bio-regulatory molecules playing a significant role in host–pathogen interactions, including influenza infection.
- miRNAs are present in numerous bodily fluids and are highly stable in these fluids. They have potential as minimally invasive disease markers. Blood, serum, saliva, and bronchial wash/lavage can be used as starting materials to detect differentially expressed miRNAs in response to influenza infection [287] what could be used in diagnostic tests and for the disease severity prognostication.
- Differential expression profiles of host miRNAs, also called the miRNAome, have been reported in vitro and in vivo with various influenza strains [287].
- Genes regulating antibody response behave differently in young and older adults [282].

All the above-mentioned findings could be used for personalization of the vaccine and vaccine dosage.

Analysis of miRNA production after influenza vaccination revealed some common patterns in the study of Nakaya et al. [283]—especially differences between the young and the elderly. miRNA production in the elderly was mainly up-regulated while that in the young was down-regulated. This miRNA study was restricted to just one year, namely vaccination in 2010. The resulting miRNA—mRNA patterns seemed to regulate interferon production—hence pathogen interference. Hence, the role for interferons noted in in Section 5.

The missing information is whether different seasonal influenza vaccines induce different miRNA responses—however given the different antigenic mix and the known differential response to influenza strains [287] this is almost certain to occur.

### 9.8. Influenza Vaccination in Coinfection and Superinfection

When analyzing pathogen interference in relation to vaccination, we must also pay attention to several very important but underestimated cases of the interference: the coinfection and superinfection in relation to a vaccine itself. There is increasing scientific attention to health and populational consequences of coinfection and superinfection. The number of research papers on this topic is growing up to 1500 papers per year [289]. However, there are unexpectedly very few research studies on the related topic concerning consequences of combinations of vaccination + infection and vaccination + superinfection. Meanwhile, in case of live attenuated vaccines, we can observe a real coinfection: live attenuated virus + wild pathogenic virus of the same or different antigenic structure (intra-species coinfection) or even—coinfection with a pathogen of different species. In case of other vaccine types (inactivated, subunit, polypeptide, RNA, etc.), in which the immune system and whole organism of vaccinated person is facing challenges which partially may resemble conditions of coinfection or superinfection in terms of necessity to develop appropriate immune response to multiple antigens in situation when vaccination already caused significant loading on the immune system and specific changes in the expression patterns of various cell types of the vaccinated person. Further complicating the picture is the immunologic legacy of multiple exposures to influenza antigens each year—from the vaccine and from wild-type viruses [290]. Besides, we should remember about the microbiome: an extremely important for our health “community of microorganisms that can usually be found living together in any given habitat”—as discussed in Section 3.7. So, any vaccination could be considered as a superinfection in relation to our microbiome.

Both these important possibilities: the coinfection and superinfection in relation to vaccination, are underrepresented in the research publications. Meanwhile, some rare publications we managed to find witness about significant influence of a vaccination on the microbial populations of the vaccinated person and potential high significance of such interference, just to mention:

A. “live attenuated influenza vaccination led to significant changes in microbial community structure, diversity, and core taxonomic membership as well as increases in the relative abundances of Staphylococcus and Bacteroides genera” [291].

B. “*S. pneumoniae* density was substantially higher in vaccine recipients (16,687 vs. 1935 gene copies per milliliter) 28 days after the first dose of Live Attenuated Influenza Vaccine (*p*  <  0.001). These findings suggest that bacterial density, and thus transmission rates among children and to people in other age groups, may rise following attenuated influenza infections” [292].

In conditions of shortage of the experimental data related to vaccination + wild virus combinations, we can roughly anticipate possible consequences of such combinations (and their frequency) using available data on coinfection and superinfection. There are some key conclusions from a very detailed review of literature on this topic [289]:

(1) The many pathogens that infect humans (e.g., viruses, bacteria, protozoa, fungal parasites, helminths) often co-occur within individuals. The true prevalence of coinfection likely exceeds one sixth of the global population.
(2) Coinfections often involve less-common pathogens.
(3) Coinfections involve a huge variety of pathogens, and most studies report negative effects on human health.
(4) The overall consequence of reported coinfections was poorer host health and enhanced pathogen abundance, compared with single infections. This is strongly supported by significant statistical differences in the reported direction of effects (*p* < 0.001).
(5) The long-term effects of coinfections can be varied and may include chronic inflammation, immunosuppression, liver fibrosis, meningitis, renal failure, rheumatic fever, etc. [293].
(6) Improved understanding of coinfection prevalence is greatly needed, partly because coinfecting pathogens can interact either directly with one another or indirectly via the host’s resources or immune system [294].
(7) Compared to infections of single pathogen species, these interactions within coinfected hosts can alter the transmission, clinical progression, and control of multiple infectious diseases [295,296].
(8) Establishing the nature and consequences of coinfection requires integrated monitoring and research of different infectious diseases, but such data are rare [297,298].
(9) Reviews of coinfection have emphasized that coinfection requires further research, especially in humans, where coinfection outnumbers single infection in many communities [289,299].
(10) To date, most disease control programs typically adopt a vertical approach to intervention, dealing with each pathogen infection in isolation. If coinfecting pathogens generally interact to worsen human health, as suggested here, control measures may need to be more integrated [289].

We also should consider the global biospheric consequences of the co-infections and super-infections (including those with attenuated vaccine strains): they influence pathogens’ ecology and evolution. Mixed infections may lead to the maintenance of genetic diversity in a host, and high levels of diversity can promote the emergence of novel genetic variants that might evolve and adapt into novel genotypes or strains, and thus, into novel diseases. Understanding about how the interactions between viruses within a host shape the evolutionary dynamics of the viral populations is needed for viral disease prevention and management [300,301].

Hence, in the conditions of widespread use of vaccines, there should be much more attention and fundamental research programs dedicated to study of consequences of the inadvertent interference (super-infection) of vaccine strain(s) with microbiome of the vaccinee in terms of possible health consequences both for the vaccinated person and exposure persons. Even more research results we expect to see concerning possible health consequences of coinfection of the vaccinated people during the period starting few days before immunization (the average latent period of infections) and ending 3 weeks after vaccination: how efficient an organism and its immunity can respond to the double challenge with vaccine and various possible wild pathogens. Additional research on various aspects of the “vaccine-superinfection” (vaccination and consequent infection with unrelated to the vaccine pathogens) are also desirable.

### 9.9. Roles for Defective Interfering Particles (DIPs and DIGs)

All the above was related to possible effects of the inter-species pathogen interference on specific and non-specific consequences of the influenza vaccination. Meanwhile, our analysis would be incomplete if not to mention a known phenomenon of intra-species pathogen interference (competition) in the field of influenza infection. This is the phenomenon of Defective Interfering Particles (DIPs), another name-Defective Interfering RNAs (DIRs). The DIPs are virions that lack a part of their genome or contains numerous genetic mutations (DIRs) that prevent wild virus RNA replication. The virus capsule in a DIP is said to retain the wild-type antigenic properties.

Most RNA viruses generate DIPs [302], and some influenza DIPs show antiviral activity against many influenza strains, including pandemic and highly pathogenic avian strains [303]. Such inhibition even extends to nonhomologous viruses such as SARS-CoV-2 via possible stimulation of innate immunity [303].

For example, coinfection of cell cultures or animals with both: DIPs and infectious wild virus cause competition on the virus RNA replication level between the DIRs and infectious virus RNA. This competition significantly reduces infectivity of the intact virus, converts potentially lethal (for mice) infection into subclinical form and induces significant immune response to the infectious virus [304]. That is why DIPs (DIRs) are studying as promising candidates for antiviral therapy and prophylaxis [303,304,305,306].

It is necessary to say that consequences of the DIPs contamination of vaccine strains are not solely positive. Previously, it was considered as a negative factor because of decrease in the yielding capacity of the vaccine strain and because DIPs are able to facilitate formation of persistent viral infections [306,307]. It is known that persistent virus infection accelerates degradation of immunity and provokes inflammation disorders which could be life-threatening in elderly. That is why consequences of the intra-species pathogen interference between the DIPs (DIGs) and intact infectious virions, as well as consequences of presence of the DIPs in influenza vaccines and their potential use for influenza prophylaxis and therapy requires additional detailed investigations and analysis [308,309,310,311].

The whole issue of DIPs/DIRs takes the whole concept of coinfection in the previous section to a new level of complexity, especially in cases of coinfection with two wild type RNA viruses. The wider implications of DIPs/DIRs to pathogen interference remains almost completely unexplored.

### 9.10. Years in Which Specific and Nonspecific Effects of Influenza Vaccination Interact

A perusal of Figure 2 [3] shows four outlying years, namely, 1988/89 and 2003/04 where influenza vaccination was associated with unusually low winter mortality and 2014/15 and 2017/18 where influenza vaccination was associated with unusually high winter mortality. Regarding the two high mortality winters both Figure 6 and Figure A1 have demonstrated unique single-year-of-age mortality patterns.

For both the unusually low/high winters researchers will need to look back at the mix of influenza strains prevalent in countries above and below the international trend line for each year [3]. This may make it possible to separate out the specific from the nonspecific effects.

### 9.11. The VE—Pathogen Interference Conundrum

As was described in Section 3 influenza vaccination occurs in an individual context. Vaccination then promotes a cascade of miRNAs modified by that context which then leads to up- and down-regulation of genes and production of various interferons. Interferons play a critical role in the regulation of immune function [312], and even re-activate dormant persistent pathogens.

This can then be followed by infection by one (or more) ‘wild’ influenzas circulating in that location at that time and the antigenic distance between the ‘wild’ influenzas and the vaccine strains then initiate a range in individual VE’s.

A significant part of vaccinated (and non-vaccinated) individuals will then be infected by one or more non-influenza winter pathogens and this infection will be moderated by the exact pathogen(s) and the individual’s response to vaccination. The likelihood of infection by different pathogens will depend on latitude, altitude, and associated local meteorological variables, including air pollution—and upon the timing of influenza vaccination in each individual with respect to the pathogens circulating at that time [15].

All steps in these processes exhibit high complexity and influence resulting VE—a central theme of this series [1,2,3].

As discussed in Section 7 the measurement of VE is subject to multiple hidden assumptions which may cumulatively lead to the lack of apparent association between the calculated VE and the effect of vaccination on EWM shown in Figure 4. A realistic estimate of influenza VE in adults was made by the Cochrane Centre for Evidence Based Medicine: “Older adults receiving the influenza vaccine may experience less influenza over a single season, from 6% to 2.4%, meaning that 30 people would need to be vaccinated with inactivated influenza vaccines to avoid one case of influenza.”—and this case may not involve hospitalization or death [117].

## 10. Further Studies

It is widely recognized that influenza vaccination is protective against influenza related hospitalization and death in persons with impaired immunity and other long-term conditions such as diabetes, etc. Given the roles of pathogen burden and pathogen interference upon different aspects of immune function, it now needs to be established as to the exact range of conditions in which influenza vaccination offers net protection for all-cause winter mortality.

Larger countries (high total deaths) with reliable data such as the USA, Brazil, Russian Federation, China, Japan, South Korea, etc., should use state/province data to conduct further targeted studies relating to altitude, latitude, meteorological variables, and the role of indoor temperature.

We also highlight that the results of the previous study regarding the unanticipated effects of influenza vaccination upon winter mortality are an international average [3]. Countries above the international trend line for each year probably experience higher pathogen interference in that year, and the reciprocal for those below.

## 11. Individual Risk

As was pointed out in the Hungarian study the risk to the individual greatly depends on the timing of vaccination and the relative mix of pathogens at that point in time [15]. The logistics of vaccinating large numbers of individuals (perhaps unnecessarily) implies that vaccination will begin early in the winter when influenza incidence is typically very low. It is also a general rule to perform vaccination in conditions when circulation of the “wild” pathogenic viruses is low to avoid the unpredictable negative consequences of simultaneous introduction of multiple antigens in an organism (wild virus + vaccine antigens). These negative effects could range from an overload and exhaustion of the immune system resources and up to hyper-reactivity (ex.-cytokine storm). A percentage of deaths from COVID are explained by vaccination of people who were already infected—at an incubation period. This caused aggravation of the disease. Hence, the earliest to be vaccinated are at the greatest risk of non-influenza infection and consequent pathogen interference. This will be further modified by the nuances of the immune response in that individual [263,264,265,266,267,313,314,315,316,317] and the miRNA(s) response to the vaccine—which remains a poorly understood area.

Unless there is a dramatic breakthrough in influenza vaccination technology it is proposed that influenza vaccination needs to be implemented as an outworking of personalized medicine rather than blanket vaccination of all persons aged 65+.

Regarding individual risk a genetic basis for mild versus severe influenza requires investigation [313,317].

An especially important study investigated which genes were involved in adverse outcomes from respiratory infections including influenza and COVID-19 [316]. The authors concluded that:

“The 166-gene signature was surprisingly conserved across all viral pandemics, including COVID-19, and a subset of 20-genes classified disease severity, inspiring the nomenclatures ViP and severe-ViP signatures, respectively. The ViP signatures pinpointed a paradoxical phenomenon wherein lung epithelial and myeloid cells mount an IL15 cytokine storm, and epithelial and NK cell senescence and apoptosis determine severity/fatality. Precise therapeutic goals could be formulated; these goals were met in high-dose SARS-CoV-2-challenged hamsters using either neutralizing antibodies that abrogate SARS-CoV-2 ACE2 engagement or a directly acting antiviral agent, EIDD-2801. IL15/IL15RA were elevated in the lungs of patients with fatal disease, and plasma levels of the cytokine prognosticated disease severity.”

The 20 ‘severe-ViP’ genes were involved in, among other aspects of health such as, DNA methylation and amyloid fiber formation [317]. DNA methylation acts to control gene expression while amyloid fiber formation is implicated in Alzheimer’s disease.

## 12. Recommendations

It is recommended that all Public Health Agencies report the “effective” alternate VE for persons who have received influenza vaccination and subsequently present with non-influenza ILI or ARI. The raw data for this calculation has been available for many years, but up to the present has not been routinely reported. This needs to be reported every season. Recalculation of historic data are possible and strongly recommended.

VE studies should be expanded to recruit far more persons aged 65+, and especially in the mid-80 s where the frequency (the mode) of death is highest, such that sufficient data are available to assess VE with age as a continuous variable.

## 13. Conclusions

This review has attempted to frame pathogen interference within a wider complex system context explored in previous papers [1,2,3]. Regarding the number of detected human pathogens we note that global warming induced melting of glaciers is releasing hundreds of new species of ancient pathogens [318]. Sampling of 21 Tibetan glaciers identified 968 candidate new species of unknown clinical significance [318]. Other areas where current knowledge is limited have been highlighted, which includes how influenza vaccination can act to alter the pathogen balance. Given the known assumption within influenza VE calculations that pathogen interference does not act as a confounder the potential for further hidden assumptions was explored. There are seeming substantial flaws in this methodology.

From the studies available, influenza vaccination seemingly precipitates complex shifts in the pathogen balance in both children and the elderly. The magnitude of such shifts varies from year-to-year. The evidence in working age adults is unclear, however, they experience a different mix of pathogens.

Influenza vaccination is clearly not universally beneficial in every winter and vaccination of persons aged 65+ without reference to their wider immune state is seemingly not recommended. Studies are required to determine which individuals, respiratory microbiota, and wider environmental/pathogen circumstances lie behind the need for, and net success of influenza vaccination in the real world of multiple pathogens.

Increasing levels of influenza vaccination do, in 40% of years, lead to an unexpected increase in excess winter mortality. This confirms the seemingly paradoxical situation whereby influenza vaccination does protect against subsequent influenza infection but is seemingly at the cost of higher susceptibility to infection by non–influenza pathogens. Further work is required to elucidate the exact immune mechanisms. Despite improvements in influenza vaccination technology over the last 80 years [257,258,259,260,261,262], specific issues seem to remain. The central issue at stake is how do we construct vaccines which avoid the seemingly unintended effects of the current types of influenza vaccines [24,58,63,64].

In hindsight, vaccines targeting the most antigenically volatile part of the influenza surface coat have inadvertently precipitated some serious unintended consequences. Alternative approaches targeting more stable surface antigens are available.

In the conditions of widespread use of vaccines, there should be much more attention and fundamental research programs dedicated to study the consequences of the inadvertent interference (coinfection) of a vaccination with the microbiome of the vaccinee in terms of possible health consequences. Further research is needed concerning possible health consequences of coinfection of the vaccinees during the period starting few days before immunization (the average latent period of infections) and ending 3 weeks after vaccination: how efficient can immunity respond to the double challenge with vaccine and various possible wild pathogenic antigens. Additional research on various aspects of superinfection (vaccination and consequent infection with unrelated to the vaccine pathogens) are also desirable.

Lastly, we need understand how influenza vaccination appears to work against pathogen interference in some years yet enhances it in others. The need for a personalized medicine approach to influenza vaccination is highlighted.

## 14. Epilogue

To put this review in context we quote from an excellent piece of investigative journalism by Jon Cohen [290]:

*“many influenza researchers are hesitant to discuss problems with the vaccine because they’re afraid of being tainted with the antivaccine brush. That’s a mistake. This immunization program has been predicated on assumptions on top of assumptions. Unless we have these discussions, we’ll never have improved vaccine options. And I don’t think it’s antivaccine to want your vaccine program to be the best that it can be”*

Danuta Skowronski, Epidemiologist, BC Centre for Disease Control, Vancouver, Canada

## Figures and Tables

**Table 1:** Number of known (detected) species of human pathogen in 2005 and 2012 [26,27], and estimated potential by 2022.

Pathogen Type	In 2005	In 2012	7-Year Increase	Potential by 2022
Bacteria	538	1003	465 (86%)	>1600
Fungi	317	447	130 (41%)	540
Helminthes	287	301	14 (5%)	305
Virus	208	274	66 (32%)	350
Protozoa	57	82	25 (44%)	100
Total	1407	2107	700 (50%)	>2895

**Table 2:** Interactions between 21 common respiratory pathogens (12 species) detected in acute respiratory illness (ARI) patients in inpatient and outpatient contexts (80% children, 20% adult) during the winter of 2005/06 (an average EWM winter) in Vancouver, Canada. Samples from nasopharyngeal wash. Different strains count as multiple pathogens. Potential interactions with influenza(s) highlighted in **bold**. Adapted from [103]. Prevalence does not add to 100% due to rounding.

Pathogen	Prevalence (%)	Enhances Infection by	Diminishes Infection by
*Neisseria meningitidis*	<1%	**Influenza B**	–
Mycoplasma pneumophilia	1%	–	–
Adenovirus (ADV 3; 4; 7; 21)	1%	–	*S. pneumoniae*; HINF 1; hMPV
Parainfluenza virus(PIV)	5%	–	*S. pneumoniae*; **Influenza A**; Rhinovirus
Influenza A	6%	–	RSV B; CVEV; Rhinovirus
Influenza B	7%	*N. meningitidis*; HINF 1	RSV A + B; CVEV; Rhinovirus
Rhinovirus	8%	CVEV	hMPV; **Influenza A + B**
Respiratory syncytial virus A (RSV A)	10%	*S. pneumoniae*	RSV B; **Influenza B**; hMPV
Respiratory syncytial virus B (RSV B)	10%	–	RSV A; hMPV; **Influenza A + B**
Human metapneumovirus (hMPV)	11%	–	RSV A + B; CVEV; Rhinovirus; PIV 3
Coxsackie/echovirus family(CVEV)	13%	Rhinovirus	**Influenza A + B**;PIV 1 + 3; hMPV
Haemophilis influenzae (HINF 1; 2; 3)	16%	*S. pneumoniae*; **Influenza B**	–
*Streptococcus pneumoniae*	20%	HINF 1 + 3	–

## Data Availability

All data used in this study is publicly available. A copy of the source data can be obtained on request from R.P.J.

## Appendix A

Figure A1, Figure A2, Figure A3 and Figure A4 relating to Section 6.6. The age-specificity in all-cause mortality, hospital admissions, and influenza confirmed deaths associated with particular influenza seasons.
